# Application of microRNA Database Mining in Biomarker Discovery and Identification of Therapeutic Targets for Complex Disease

**DOI:** 10.3390/mps4010005

**Published:** 2020-12-30

**Authors:** Jennifer L. Major, Rushita A. Bagchi, Julie Pires da Silva

**Affiliations:** Department of Medicine, Division of Cardiology, University of Colorado Anschutz Medical Campus, Aurora, CO 80045, USA; jennifer.major@cuanschutz.edu

**Keywords:** microRNA, HMDD v3.2, ischemia-reperfusion, obesity, coronary artery disease, stroke

## Abstract

Over the past two decades, it has become increasingly evident that microRNAs (miRNA) play a major role in human diseases such as cancer and cardiovascular diseases. Moreover, their easy detection in circulation has made them a tantalizing target for biomarkers of disease. This surge in interest has led to the accumulation of a vast amount of miRNA expression data, prediction tools, and repositories. We used the Human microRNA Disease Database (HMDD) to discover miRNAs which shared expression patterns in the related diseases of ischemia/reperfusion injury, coronary artery disease, stroke, and obesity as a model to identify miRNA candidates for biomarker and/or therapeutic intervention in complex human diseases. Our analysis identified a single miRNA, hsa-miR-21, which was casually linked to all four pathologies, and numerous others which have been detected in the circulation in more than one of the diseases. Target analysis revealed that hsa-miR-21 can regulate a number of genes related to inflammation and cell growth/death which are major underlying mechanisms of these related diseases. Our study demonstrates a model for researchers to use HMDD in combination with gene analysis tools to identify miRNAs which could serve as biomarkers and/or therapeutic targets of complex human diseases.

## 1. Introduction

Although the majority of the genome is transcribed, only a small percentage of it is translated into protein-coding RNA, the remaining being generally classified as non-coding RNAs. Based on size, noncoding RNA can be divided into short noncoding RNA (including microRNA, siRNA, snoRNA, piRNA, and pRNAs) and long non-coding RNA (reviewed in [1]). The discovery of these novel molecules in the early 1990s and their biological activity in the early 2000s shattered the central dogma and led to an explosion of research revealing their importance in numerous disease pathophysiologies [2,3,4,5].

Perhaps the most broadly studied of the short non-coding RNAs are microRNAs (miRNA). MiRNAs are ~22 nt long RNA molecules which act as a guide for regulatory proteins to bind to messenger RNAs (mRNA) and other non-coding RNAs via a complementary seed sequence (~6–8 nt) [6]. MiRNAs are transcribed by RNA polymerase II creating a hairpin structured pre-microRNA which is cleaved into two mature miRNAs, one from the 5′ strand, and the other from the 3′ strand. In most cases, one strand is much more prevalent and biologically active than the other, which is referred to as the guide strand while the other is referred to as the passenger strand. When a mature miRNA binds to an mRNA through its seed sequence, this creates an RNA duplex which is recognized by and forms a complex with the RNA inducing silencing complex (RISC) proteins. In the classical sense, RISC formation in the cytosol can lead to the degradation of the target mRNA and/or inhibition of its translation. More recently, it has become evident that miRNAs and the RISC complex can also be detected in the nucleus where they can regulate transcriptional activation, RNA processing events such as alternative splicing, and ribosome biogenesis [7]. Moreover, miRNAs can be secreted from the cell and act as endocrine and paracrine signaling molecules, making them a tantalizing target for disease biomarkers [8].

Given their importance in disease pathology and their potential as biomarkers, numerous miRNA databases have been created detailing their sequences, tissue expression, and potential targets. These publicly available tools can be mined to identify miRNAs which have been identified in human diseases as well as which ones share a common signature among related diseases. We currently live in an era of ‘big data’—information from RNA-seq, ChIP-seq, and proteomics are becoming the norm. Scientists have compiled massive amounts of data which are available through public repositories waiting to be mined. Here, we utilize such an existing database, the Human microRNA Disease Database (HMDD), to identify miRNAs which are related to complex human diseases. While this is not a novel approach in its entirety, this manuscript provides a generalized outline for using established public databases and analysis tools to identify and develop research hypotheses.

**Figure 1:**
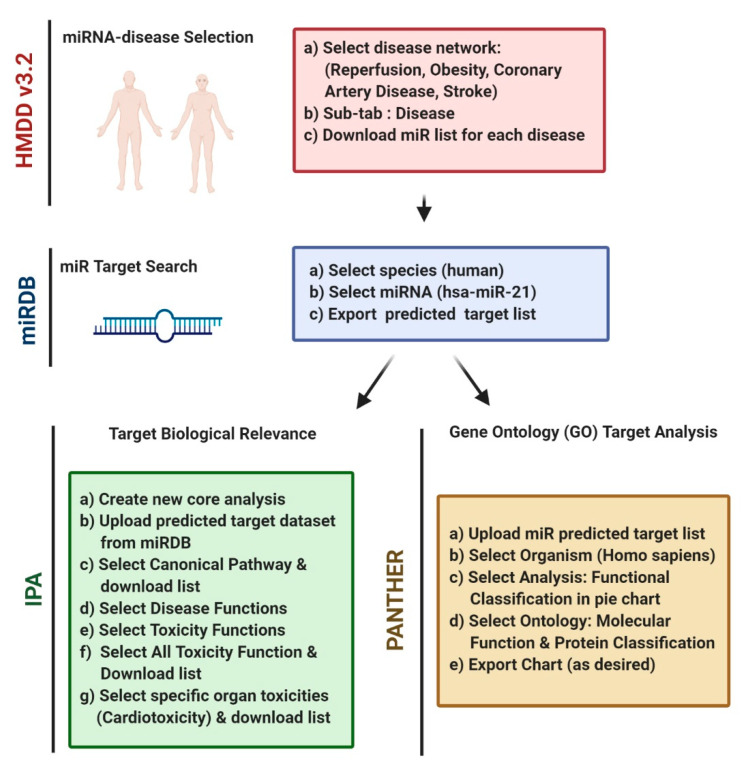
Workflow for analysis of miRNAs in human disease. Analysis begins with the identification of a disease network with shared microRNAs using Human MicroRNA Disease Database (HMDD) (1). Target mRNAs for the microRNAs identified in Part 1 are predicted using microRNA prediction database miRDB. Only targets with a prediction score >94 should be used for further analysis (2). Predicted target lists generated in Part 2 are then uploaded to Protein ANalysis THrough Evolutionary Relationships (PANTHER) and Ingenuity Pathway Analysis (IPA) for further analysis (3). This figure was created using Biorender.com.

For the purpose of this manuscript, we sought to identify miRNAs related to cardiovascular disease (CVD), which is the number one cause of nosocomial death in the world [9]. Among the 17.9 million worldwide deaths caused by CVD in 2016, approximately 85% of them were due to stroke and myocardial infarction (MI), which cause ischemia/reperfusion (I/R) injury to the heart and brain, respectively. Ischemia is defined as the restriction of blood flow to tissue, which results in injury/cell death to those tissues which do not receive the oxygen and nutrients required for cellular metabolism [10]. Paradoxically, when blood flow is restored to tissues following ischemia (reperfusion), the rapid re-introduction of oxygen initiates the production of reactive oxygen species (ROS) which can lead to a dangerous cascade of cellular injury, inflammation, and cell death [11,12]. We sought to use I/R injury and its associated diseases as a model for how to identify novel and critical miRNAs involved in complex diseases using the Human microRNA Disease Database version 3.2 (HMDD v3.2) and well-established bioinformatics packages (Figure 1).

## 2. Materials and Methods

### 2.1. Human microRNA Disease Database Version 3.2

Human microRNA Disease Database version 3.2 (HMDD v3.2) (https://www.cuilab.cn/hmdd; [13,14,15]) regrouped 35,547 miRNA-disease association entries which include 1206 miRNA genes, 893 diseases from 19,280 papers. HMDD v3.2 was used to identify four diseases of interest known to be linked by miRNA (obesity, I/R injury, coronary artery disease, and stroke).

### 2.2. Prediction of hsa-miRNA-21 Targets & Functional Analyses

The online MicroRNA Target Prediction database (miRDB) (http://mirdb.org; [16]), based on support vector machines (SVMs) and high-throughput training datasets, was used for prediction of the mRNA targets of hsa-miR-21-5p and hsa-miR-21-3p. The top 20 targets (target score >94) were used for functional analysis. The predicted targets have target prediction scores between 50–100. A predicted target with prediction score >80 is most likely to be relevant. Gene ontology (GO) is a common method to collect information about gene product function [17]. PANTHER (Protein ANalysis THrough Evolutionary Relationships) method is based on multiple sequence alignment, hidden Markov model, and family tree [18]. GO categorization using PANTHER (http://www.pantherdb.org; [19]) was performed to investigate the molecular function and protein class GO terms associated with hsa-miR-21-5p and hsa-miR-21-3p targets. Ingenuity Pathway Analysis (IPA) is a web platform which runs upstream regulator analysis (URA), mechanistic networks (MN), downstream effects analysis (DEA), and causal network analysis (CNA) algorithms in the back end [20]. IPA and PANTHER platforms were used to investigate molecular pathways and toxicity functions associated with hsa-miR-21-5p and hsa-miR-21-3p target genes.

## 3. Results

The Human MicroRNA Disease Database was created in 2007 by Dr. Cingua Qui’s lab to allow researchers to discover miRNAs associated with diseases based on scientific evidence (i.e., publications) [15]. Due to a surge in new data, the authors have made over 30 updates to the database in the last twelve years including a second version (HMDD v2.0) in 2014 [14] and the latest version (HMDD v3.0) was released in 2019 with double the amount of miRNAs classified into six main categories based on experimental evidence (circulation, tissue, genetics, epigenetics, targets, or other) [13].

**Figure 2:**
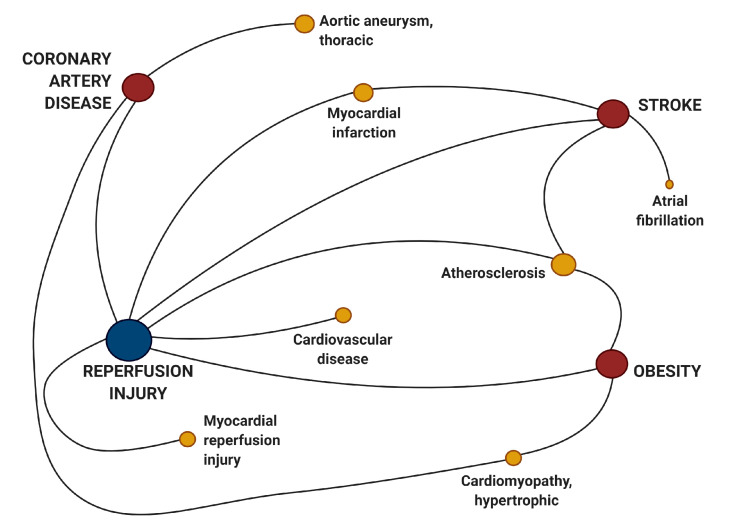
Visualization of hsa-miR associated with ischemia/reperfusion injury. Ischemia/reperfusion injury network was created via the integration of hsa-miR–disease association data with the categories “genetics”, “epigenetics”, “circulating”, and “target” inputted into the Human microRNA Disease Database. This figure was created using Biorender.com.

To begin our research, we used HMDD v3.2′s new visualization tools to find other diseases which shared deregulated miRNAs in common with I/R injury. As anticipated, we found I/R injury shared many miRNAs in common with those which are known to cause or be associated with stroke and coronary artery disease (CAD) (Figure 2). Perhaps more interesting was the strong connection to obesity which is one of the major risk factors for CVD including CAD, stroke, and MI.

Based on positive causality (genetics), the program identified eight hsa-miRNAs, but only one, hsa-miR-21, was linked with all four diseases (Table 1). When considering hsa-miRNAs which were deregulated in the four diseases (but not causally linked), we found that hsa-miR-21 can also be detected in circulation in each disease state (Table S1). A variety of other hsa-miRNAs were detected in the circulation (Table S1), but hsa-miR-21 was the only miRNA consistently detected in all four diseases, thereby making it an attractive candidate for both targeted therapeutics and biomarker detection, thus, it became the focus of our further analysis.

To gauge the mechanism by which hsa-miR-21 could potentially regulate the four associated diseases, we used the online database miRDB to identify putative mRNA targets. A total of 469 targets for hsa-miR-21-5p and 594 targets for hsa-miR-21-3p were identified using miRDB (Tables S2 and S3). Based on miRDB target score (target score >94), the top twenty targets for hsa-miR21-5p and hsa-miR21-3p are listed in Table 2 and Table 3, respectively.

**Figure 3:**
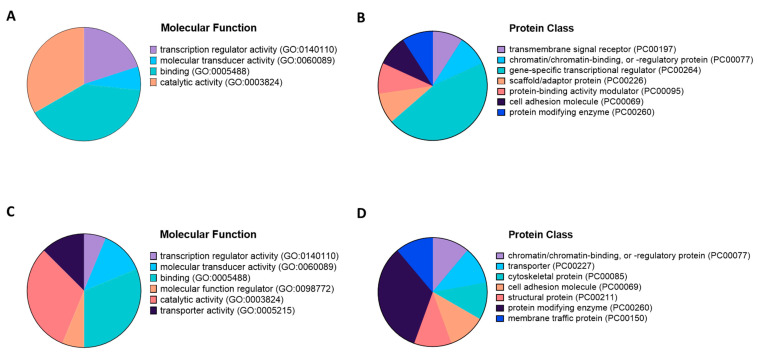
Categorization of gene ontology (GO) annotations of hsa-miR-21-5p and hsa-miR-21-3p with PANTHER. Graphical representation of the molecular function (**A**,**C**) and protein class (**B**,**D**) GO terms for the top twenty targets of hsa-miR-21-5p (**A**,**B**) and hsa-miR-21-3p (**C**,**D**).

To begin characterization of the top target gene products, we defined their molecular function and protein class GO terms using PANTHER (Figure 3). The top targets for hsa-miR21-5p could be grouped into four molecular function categories with 40% “protein binding”, 33% “catalytic activity”, 15% “transcriptional activators”, and 5% “molecular transducer activity” (Figure 3A). Almost 50% of the hsa-miR-215p targets were classified as proteins with “gene-specific transcriptional regulators” (Figure 3B). Targets for hsa-miR-21-3p were largely included in the functional classes of “molecular function regulator” and “catalytic activity”, with 31% falling into the protein class of “protein modifying enzyme” (Figure 3C,D).

To further characterize the biological processes and pathologies which might be deregulated by hsa-miR-21′s target genes, we utilized PANTHER and IPA to score canonical pathways related to both hsa-miR-21 strands. The top targets for hsa-miR-21-5p were included in many canonical pathways related to inflammation and cell death such as “Neuroinflammation Signaling Pathway”, “Natural Killer Cell Signaling”, “Necroptosis Signaling Pathway”, and “Apoptosis Signaling” (Table 4). Almost all hsa-miR-21-5p targets were related to the major molecular regulators of inflammation and cell death, interleukin 12 (IL12A) and the Fas ligand (FASLG), which are common pathways of I/R injury.

The top targets of hsa-miR-21-3p were related to mitogen activated protein kinase canonical pathways (MAPK), in particular MAP2K4 and MAP3K1 (Table 5), both of which are major regulators of cellular signaling that regulate cell growth and death.

According to IPA analysis, both hsa-miR-21-5p and hsa-miR-21-3p targets were significantly enriched in toxicity functions related to cardiotoxicity (Table 6 and Table 7). Considering that hsa-miR-21 is well recognized to be upregulated in CVD this is not surprising (reviewed in [21]). Taking all toxicity function terms into account liver and kidney toxicities were also enriched suggesting that hsa-miR-21 and its targets could also play major roles in other organs (Tables S4 and S5).

## 4. Discussion and Conclusions

I/R injuries are common pathologies of many cardiovascular conditions such as myocardial infarction, stroke, and post-cardiac arrest syndrome, which can have disastrous effects on the tissue affected and the body as a whole. While these injuries occur and induce inflammation locally after revascularization, signals such as ROS can infiltrate the bloodstream, causing inflammation and damage in remote organs [10]. Our results demonstrate that many of the miRNAs detected in I/R, CAD, stroke, and obesity can be detected in circulation and could also have an effect on remote organs. This indicates that in addition to serving as potential biomarkers for said diseases, these miRNAs which are co-expressed in related pathologies could be connected on a causal level which has yet to be demonstrated.

Obesity is a major risk factor for CAD, I/R, and stroke (among many other diseases). Adipose tissue is important for energy storage and for organ insulation, but also serves as an endocrine organ which can synthesize compounds which regulate homeostasis [22]. Thus, it is not a great leap to suggest that some circulating miRNAs such as hsa-miR-21 may be released by adipose tissue, thereby contributing to the associated I/R/CAD/stroke risks. It was recently demonstrated in a small study of human patients that serum hsa-miR-21 level was significantly higher in patients with heart failure, suggesting that hsa-miR-21 could serve as a promising biomarker for heart failure with high correlation between circulating hsa-miR-21 and prognosis and re-hospitalization rates [23].

MiR-21 expression is widely known to be upregulated in cardiovascular disease and obesity. Interestingly, while its expression appeared to have a beneficial effect against ischemic injury in the murine heart [24,25], it is generally recognized to have negative impact on vascular injury/lesion formation via its action in dedifferentiated vascular smooth muscle cells [26], and could induce cardiac pathological hypertrophy in vitro (fibroblasts, cardiomyocytes) and in vivo [27,28]. These discrepancies highlight the importance of taking a systemic approach when treating disease, especially complex ones such as obesity and its associated risks of CVD. It is possible that while acute upregulation of miR-21 after ischemic injury might be beneficial, long-term expression could be linked to cardiac hypertrophy and obesity which are then risk factors for MI/stroke.

The top target pathway for hsa-miR-21-5p was the pro-inflammatory IL12A, which has been linked to increased arterial stiffness associated with early atherosclerosis in healthy human patients which could potentially be a risk factor for future stroke/I/R [29]. It has also been suggested that rno-miR-21-5p could be a biomarker for cardiac inflammation [30]. Pathway analysis of the top hsa-miR-21-3p targets revealed that the highest ranked canonical pathways were MAP2K4 and MAP3K1 which are master regulators of cardiac pathological growth [31,32]. While miR-21 was the only miRNA evidenced to be a causal factor in all four disease processes, there are a number of other miRNAs which are deregulated in the diseases but have yet to be causally related such as hsa-miR-122 and hsa-miR-146a [33,34,35]. This group of miRNAs are likely to be useful in uncovering novel pathways of regulation of such complex diseases.

There are several other publicly available human miR databases such as ExcellmiRDB [36], IntmiR [37], miR2Disease [38], PhenomiR [39], and miRsig [40], but none are as robust in scope as HMDD v3.2. HMDD undergoes regular updates which allows users to the access most recent curated entries for the human miRs in all tissues, while others are limited to specific diseases and/or tissues. Moreover, HMDD users are able to access multiple features for analyses through easy to access front end—a key aspect not available with most other similar platforms. For example, causality which is based on direct experimental evidence, is a unique feature of the HMDD database, making it a primordial tool for studying miRNA-disease associations.

Over the past decade, it has become increasingly apparent that miRNAs play important roles in human disease through their direct regulation or protein coding mRNA. With this increased understanding, researchers are creating miRNA mimics and antagomiRs (to inhibit miRNA function) which can be delivered to patients to modulate gene expression in disease states. There are currently several miRNA clinical trials in phase one and two underway for the treatment of various cancers, as well as, one utilizing miR-21 for the treatment of patients with Alport syndrome (NCT02855268-phase 2) [41]. The results of this study will be particularly interesting given the wide variety of action for hsa-miR-21. More immediately promising is the use of miRNAs for diagnostics and biomarkers. In fact, there are hundreds of clinical studies recruiting patients for miRNA identification and detection in human diseases (clinicaltrials.gov). Currently there are several miRNA panels available for diagnostic use in human patients for a variety of cancers as well as one panel for CVD [42].

Our study demonstrates a method in which researchers can mine miRNA databases such as HMDD to find miRNAs associated with their disease of interest and how they might impact other tissues and pathologies. These publicly available resources are underestimated by the scientific community for exploring new avenues to identify potential disease biomarkers and therapeutic targets on a systemic level.

## Figures and Tables

**Table 1:** miRNAs which are causally associated with ischemia/reperfusion injury, stroke, coronary artery disease, and obesity in humans. Eight hsa-miRs were identified using the term “genetics” as input in HMDD. CAD, coronary artery disease; HMDD, Human microRNA Disease Database (v3.2).

**Genetics I/R Injury**	Targets CAD	hsa-miR-21
		hsa-miR-221
	Targets I/R injury	hsa-miR-146a
		hsa-miR-21
		hsa-miR-214
		hsa-miR-497
	Targets Stroke	hsa-miR-146a
**Genetics Obesity**	Targets CAD	hsa-miR-21
		hsa-miR-222
	Targets Stroke	hsa-miR-155
	Targets I/R injury	hsa-miR-21

**Table 2:** Top 20 gene targets of hsa-miR-21-5p. Identification of the predicted targets of hsa-miR-21-5p with a prediction score >94 using miRDB.

Target Rank	Target Score	miRNA Name	Gene Symbol	Gene Description
1	99	hsa-miR-21-5p	YOD1	YOD1 deubiquitinase
2	99	hsa-miR-21-5p	FASLG	Fas ligand
3	99	hsa-miR-21-5p	PRDM11	PR/SET domain 11
4	99	hsa-miR-21-5p	VCL	vinculin
5	99	hsa-miR-21-5p	ZNF367	zinc finger protein 367
6	98	hsa-miR-21-5p	SKP2	S-phase kinase associated protein 2
7	98	hsa-miR-21-5p	TGFBI	transforming growth factor beta induced
8	97	hsa-miR-21-5p	IL12A	interleukin 12A
9	97	hsa-miR-21-5p	RAB6D	RAB6D, member RAS oncogene family
10	97	hsa-miR-21-5p	ADGRG2	adhesion G protein-coupled receptor G2
11	97	hsa-miR-21-5p	RALGPS2	Ral GEF with PH domain and SH3 binding motif 2
12	97	hsa-miR-21-5p	PLAG1	PLAG1 zinc finger
13	97	hsa-miR-21-5p	RBPJ	recombination signal binding protein for IgK J region
14	97	hsa-miR-21-5p	PELI1	pellino E3 ubiquitin protein ligase 1
15	97	hsa-miR-21-5p	CREBRF	CREB3 regulatory factor
16	97	hsa-miR-21-5p	KRIT1	KRIT1, ankyrin repeat containing
17	96	hsa-miR-21-5p	SCML2	Scm polycomb group protein like 2
18	96	hsa-miR-21-5p	RSAD2	radical S-adenosyl methionine domain containing 2
19	96	hsa-miR-21-5p	PBRM1	polybromo 1
20	96	hsa-miR-21-5p	GATAD2B	GATA zinc finger domain containing 2B

**Table 3:** Top 20 gene targets of hsa-miR-21-3p. Identification of the predicted targets of hsa-miR-21-3p with a prediction score >94 using miRDB.

Target Rank	Target Score	miRNA Name	Gene Symbol	Gene Description
1	99	hsa-miR-21-3p	STK38L	serine/threonine kinase 38 like
2	98	hsa-miR-21-3p	PCDH19	protocadherin 19
3	96	hsa-miR-21-3p	LAMP1	lysosomal associated membrane protein 1
4	96	hsa-miR-21-3p	GRIA2	glutamate ionotropic receptor AMPA type subunit 2
5	96	hsa-miR-21-3p	TOGARAM1	TOG array regulator of axonemal microtubules 1
6	96	hsa-miR-21-3p	ATP1B1	ATPase Na^+^/K^+^ transporting subunit beta 1
7	96	hsa-miR-21-3p	TSC22D2	TSC22 domain family member 2
8	96	hsa-miR-21-3p	NAP1L5	nucleosome assembly protein 1 like 5
9	95	hsa-miR-21-3p	UBE4B	ubiquitination factor E4B
10	95	hsa-miR-21-3p	ZNF326	zinc finger protein 326
11	95	hsa-miR-21-3p	CDK8	cyclin dependent kinase 8
12	94	hsa-miR-21-3p	MAP2K4	mitogen-activated protein kinase kinase 4
13	94	hsa-miR-21-3p	AKAP11	A-kinase anchoring protein 11
14	94	hsa-miR-21-3p	GPM6A	glycoprotein M6A
15	94	hsa-miR-21-3p	MAP3K1	mitogen-activated protein kinase kinase kinase 1
16	94	hsa-miR-21-3p	PARD3B	par-3 family cell polarity regulator beta
17	94	hsa-miR-21-3p	ALCAM	activated leukocyte cell adhesion molecule
18	94	hsa-miR-21-3p	FOXO3	forkhead box O3
19	94	hsa-miR-21-3p	FYTTD1	forty-two-three domain containing 1
20	94	hsa-miR-21-3p	PHYHIPL	phytanoyl-CoA 2-hydroxylase interacting protein like

**Table 4:** Canonical pathways associated with top 20 hsa-miR-21-5p target genes. Canonical pathways identified via Ingenuity Pathway Analysis for the top 20 gene targets of hsa-miR-21-5p are reported.

Canonical Pathways	Molecules
Crosstalk between Dendritic Cells and Natural Killer Cells	FASLG, IL12A
Altered T Cell and B Cell Signaling in Rheumatoid Arthritis	FASLG, IL12A
PD-1, PD-L1 cancer immunotherapy pathway	IL12A, SKP2
Type I Diabetes Mellitus Signaling	FASLG, IL12A
Airway Pathology in Chronic Obstructive Pulmonary Disease	FASLG, IL12A
Role of Pattern Recognition Receptors in Recognition of Bacteria and Viruses	FASLG, IL12A
Necroptosis Signaling Pathway	FASLG, PELI1
HMGB1 Signaling	FASLG, IL12A
Hepatic Cholestasis	FASLG, IL12A
Natural Killer Cell Signaling	FASLG, IL12A
Differential Regulation of Cytokine Production in Macrophages and T Helper Cells by IL-17A and IL-17F	IL12A
Role of Lipids/Lipid Rafts in the Pathogenesis of Influenza	RSAD2
Differential Regulation of Cytokine Production in Intestinal Epithelial Cells by IL-17A and IL-17F	IL12A
Tumoricidal Function of Hepatic Natural Killer Cells	FASLG
Estrogen-mediated S-phase Entry	SKP2
Systemic Lupus Erythematosus In B Cell Signaling Pathway	FASLG, IL12A
Neuroinflammation Signaling Pathway	FASLG, IL12A
Cytotoxic T Lymphocyte-mediated Apoptosis of Target Cells	FASLG
Notch Signaling	RBPJ
Antiproliferative Role of TOB in T Cell Signaling	SKP2

**Table 5:** Canonical pathways associated with top 20 hsa-miR-21-3p target genes. Canonical pathways identified via Ingenuity Pathway Analysis for the top 20 gene targets of hsa-miR-21-3p are reported.

Canonical Pathways	Molecules
EGF Signaling	MAP2K4, MAP3K1
Toll-like Receptor Signaling	MAP2K4, MAP3K1
NRF2-mediated Oxidative Stress Response	MAP2K4, MAP3K1
Glutamate Receptor Signaling	GRIA2
FGF Signaling	MAP3K1
Apoptosis Signaling	MAP2K4
p38 MAPK Signaling	MAP2K4
Inhibition of Angiogenesis by TSP1	MAP2K4
Integrin Signaling	MAP2K4
IGF-1 Signaling	FOXO3
PI3K/AKT Signaling	FOXO3
Huntington’s Disease Signaling	MAP2K4
TNFR2 Signaling	MAP2K4, MAP3K1
April Mediated Signaling	MAP2K4, MAP3K1
B Cell Activating Factor Signaling	MAP2K4, MAP3K1
TNFR1 Signaling	MAP2K4, MAP3K1
CD27 Signaling in Lymphocytes	MAP2K4, MAP3K1
Pyridoxal 5′-phosphate Salvage Pathway	CDK8, MAP2K4
Ceramide Signaling	MAP2K4, MAP3K1
RANK Signaling in Osteoclasts	MAP2K4, MAP3K1

**Table 6:** Cardiotoxicity functions of hsa-miR-21-5p targets. Cardiotoxicity functions identified via Ingenuity Pathway Analysis of the top 20 target genes of hsa-miR-21-5p are reported.

Category	Molecules
Cardiac Dilation	VCL
Cardiac Enlargement	FASLG, VCL
Heart Failure	RBPJ, VCL
Cardiac Hypoplasia	PBRM1, RBPJ
Cardiac Dysfunction	FASLG, RBPJ, VCL
Cardiac Arrhythmia	VCL
Cardiac Inflammation	RBPJ
Tachycardia	VCL

**Table 7:** Cardiotoxicity functions of hsa-miR-21-3p targets. Cardiotoxicity functions identified via Ingenuity Pathway Analysis of the top 20 target genes of hsa-miR-21-3p are reported.

Category	Molecules
Cardiac Necrosis/Cell Death	FOXO3, MAP2K4, MAP3K1, UBE4B
Heart Failure	MAP2K4, MAP3K1, UBE4B
Cardiac Congestive Cardiac Failure	MAP3K1, UBE4B
Cardiac Enlargement	FOXO3, MAP2K4, UBE4B
Cardiac Arrhythmia	ATP1B1, FOXO3
Cardiac Inflammation	MAP3K1
Cardiac Infarction	FOXO3
Cardiac Dilation	FOXO3

## Data Availability

The data presented in this study are available in “Application of microRNA Database Mining in Biomarker Discovery and Identification of Therapeutic Targets for Complex Disease” and in Supplementary material.

## Supplementary Materials

The following are available online at https://www.mdpi.com/2409-9279/4/1/5/s1, Table S1: List of all hsa-miRs identified by Human microRNA Disease Database (HMDD; v3.2) analysis. Table S2: List of all hsa-miR-21-5p targets. Table S3: List of all hsa-miR-21-3p targets. Table S4: Toxicity functions reported for hsa-miR-21-5p. Table S5: Toxicity functions reported for hsa-miR-21-3p.
