# Supplementary material for: Application of microRNA Database Mining in Biomarker Discovery and Identification of Therapeutic Targets for Complex Disease
Source: Methods Protoc. 2020 Dec 30;4(1):5. doi: 10.3390/mps4010005 (PMC7838776; doi:10.3390/mps4010005)
Supplement: Supplementary file 1 [file mps-04-00005-s001.pdf]

methods and protocols

MDPI

Article

Application of microRNA Database Mining in Biomarker Discovery and Identification of Therapeutic Targets for Complex Disease

Jennifer L. Major, Rushita A. Bagchi * and Julie Pires da Silva *

Department of Medicine, Division of Cardiology, University of Colorado Anschutz Medical Campus, Aurora, CO 80045, USA; jennifer.major@cuanschutz.edu
* Correspondence: rushita.bagchi@cuanschutz.edu (R.A.B.); julie.piresdasilva@cuanschutz.edu (J.P.d.S.)

Supplementary Tables

Table 1. List of all hsa-miRs identified by Human microRNA Disease Database (HMDD; v3.2) analysis. hsa-miRs were identified using the term "genetics" and "circulating" as input in HMDD.

**Genetics CAD**	**Targets CAD**	hsa-miR-1
	**Targets IR injury**	hsa-miR-423
	**Targets Obesity**	hsa-miR-499
	**Circulating Obesity**	hsa-miR-146a
		hsa-miR-423
	**Circulating CAD**	hsa-miR-146a
		hsa-miR-149
		hsa-miR-499
	**Circulating IR Injury**	hsa-miR-146a
**Genetics Stroke**	**Circulating Obesity**	hsa-miR-122
	**Circulating CAD**	hsa-miR-122
	**Circulating Stroke**	hsa-miR-122
**Genetics Obesity**	**Circulating Stroke**	hsa-miR-26b
**Circulating Obesity**	**Targets CAD**	hsa-miR-17
		hsa-miR-223
		hsa-miR-340
		hsa-miR-34a
		hsa-miR-92a
	**Targets IR injury**	hsa-miR-126
		hsa-miR-21
		hsa-miR-423
	**Targets Obesity**	hsa-miR-126
		hsa-miR-143
		hsa-miR-21
		hsa-miR-223
		hsa-miR-34a
**Circulating CAD**	**Targets CAD**	hsa-miR-17
		hsa-miR-223
		hsa-miR-92a
	**Targets IR injury**	hsa-miR-126
		hsa-miR-155
		hsa-miR-21
	**Targets Obesity**	hsa-miR-126
		hsa-miR-145
		hsa-miR-21
		hsa-mir-223
		hsa-mir-499
		hsa-mir-574
**Circulating IR injury**	**Targets IR injury**	hsa-mir-21
	**Targets Obesity**	hsa-mir-21
**Circulating Stroke**	**Targets CAD**	hsa-mir-22
	**Targets IR injury**	hsa-mir-133a
		hsa-mir-155
		hsa-mir-21
	**Targets Obesity**	hsa-mir-145
		hsa-mir-146b
		hsa-mir-21
		hsa-mir-29b

Table 2. List of all hsa-miR-21-5p targets. Identification of all the predicted targets of hsa-miR-21-5p using miRDB.

Target Rank	Target Score	miRNA Name	Gene Symbol	Gene Description
1	99	hsa-miR-21-5p	YOD1	YOD1 deubiquitinase
2	99	hsa-miR-21-5p	FASLG	Fas ligand
3	99	hsa-miR-21-5p	PRDM11	PR/SET domain 11
4	99	hsa-miR-21-5p	VCL	vinculin
5	99	hsa-miR-21-5p	ZNF367	zinc finger protein 367
6	98	hsa-miR-21-5p	SKP2	S-phase kinase associated protein 2
7	98	hsa-miR-21-5p	TGFBI	transforming growth factor beta induced
8	97	hsa-miR-21-5p	IL12A	interleukin 12A
9	97	hsa-miR-21-5p	RAB6D	RAB6D, member RAS oncogene family
10	97	hsa-miR-21-5p	ADGRG2	adhesion G protein-coupled receptor G2
11	97	hsa-miR-21-5p	RALGPS2	Ral GEF with PH domain and SH3 binding motif 2
12	97	hsa-miR-21-5p	PLAG1	PLAG1 zinc finger
13	97	hsa-miR-21-5p	RBPJ	recombination signal binding protein for immunoglobulin kappa J region
14	97	hsa-miR-21-5p	PELI1	pellino E3 ubiquitin protein ligase 1
15	97	hsa-miR-21-5p	CREBRF	CREB3 regulatory factor
16	97	hsa-miR-21-5p	KRIT1	KRIT1, ankyrin repeat containing
17	96	hsa-miR-21-5p	SCML2	Scm polycomb group protein like 2
18	96	hsa-miR-21-5p	RSAD2	radical S-adenosyl methionine domain containing 2
19	96	hsa-miR-21-5p	PBRM1	polybromo 1
20	96	hsa-miR-21-5p	GATAD2B	GATA zinc finger domain containing 2B
21	95	hsa-miR-21-5p	SPRY1	sprouty RTK signaling antagonist 1
22	95	hsa-miR-21-5p	PLEKHA1	pleckstrin homology domain containing A1
23	95	hsa-miR-21-5p	FGF18	fibroblast growth factor 18
24	95	hsa-miR-21-5p	PPP1R3B	protein phosphatase 1 regulatory subunit 3B
25	94	hsa-miR-21-5p	YAP1	Yes associated protein 1
26	94	hsa-miR-21-5p	GPATCH2L	G-patch domain containing 2 like
27	94	hsa-miR-21-5p	STAT3	signal transducer and activator of transcription 3
28	94	hsa-miR-21-5p	BCL7A	BCL7A, BAF complex component
29	94	hsa-miR-21-5p	SKI	SKI proto-oncogene
30	94	hsa-miR-21-5p	FAM13A	family with sequence similarity 13 member A
31	94	hsa-miR-21-5p	MALT1	MALT1 paracaspase
32	93	hsa-miR-21-5p	ZBTB41	zinc finger and BTB domain containing 41
33	93	hsa-miR-21-5p	KDM7A	lysine demethylase 7A
34	93	hsa-miR-21-5p	MBNL3	muscleblind like splicing regulator 3
35	93	hsa-miR-21-5p	CCL1	C-C motif chemokine ligand 1
36	93	hsa-miR-21-5p	NKIRAS1	NFKB inhibitor interacting Ras like 1
37	93	hsa-miR-21-5p	TIAM1	T cell lymphoma invasion and metastasis 1
38	93	hsa-miR-21-5p	OSR1	odd-skipped related transcription factor 1
39	93	hsa-miR-21-5p	KLF3	Kruppel like factor 3
40	93	hsa-miR-21-5p	PAN3	poly(A) specific ribonuclease subunit PAN3
41	92	hsa-miR-21-5p	PDCD4	programmed cell death 4
42	92	hsa-miR-21-5p	AKAP12	A-kinase anchoring protein 12
43	92	hsa-miR-21-5p	GID4	GID complex subunit 4 homolog
44	92	hsa-miR-21-5p	HSD17B4	hydroxysteroid 17-beta dehydrogenase 4
45	92	hsa-miR-21-5p	PDZD2	PDZ domain containing 2
46	92	hsa-miR-21-5p	CPEB3	cytoplasmic polyadenylation element binding protein 3
47	92	hsa-miR-21-5p	CASKIN1	CASK interacting protein 1
48	92	hsa-miR-21-5p	MAP3K1	mitogen-activated protein kinase kinase kinase 1
49	92	hsa-miR-21-5p	UBE2D3	ubiquitin conjugating enzyme E2 D3
50	91	hsa-miR-21-5p	NTF3	neurotrophin 3

51	91	hsa-miR-21-5p	TIMP3	TIMP metallopeptidase inhibitor 3
52	91	hsa-miR-21-5p	RECK	reversion inducing cysteine rich protein with kazal motifs
53	91	hsa-miR-21-5p	CCL20	C-C motif chemokine ligand 20
54	91	hsa-miR-21-5p	JAG1	jagged 1
55	91	hsa-miR-21-5p	ANGPTL5	angiopoietin like 5
56	91	hsa-miR-21-5p	PPP1R3A	protein phosphatase 1 regulatory subunit 3A
57	91	hsa-miR-21-5p	BCL11B	BCL11B, BAF complex component
58	90	hsa-miR-21-5p	BTG2	BTG anti-proliferation factor 2
59	90	hsa-miR-21-5p	LRRC57	leucine rich repeat containing 57
60	90	hsa-miR-21-5p	NFIA	nuclear factor I A
61	90	hsa-miR-21-5p	MPRIP	myosin phosphatase Rho interacting protein
62	90	hsa-miR-21-5p	SLC30A10	solute carrier family 30 member 10
63	90	hsa-miR-21-5p	SYT15	synaptotagmin 15
64	90	hsa-miR-21-5p	MEI4	meiotic double-stranded break formation protein 4
65	90	hsa-miR-21-5p	GLCCI1	glucocorticoid induced 1
66	90	hsa-miR-21-5p	KLHL15	kelch like family member 15
67	90	hsa-miR-21-5p	CFAP300	cilia and flagella associated protein 300
68	90	hsa-miR-21-5p	FAM3C	family with sequence similarity 3 member C
69	90	hsa-miR-21-5p	EPM2A	EPM2A, laforin glucan phosphatase
70	90	hsa-miR-21-5p	SPRY2	sprouty RTK signaling antagonist 2
71	89	hsa-miR-21-5p	RASA1	RAS p21 protein activator 1
72	89	hsa-miR-21-5p	KDM1B	lysine demethylase 1B
73	89	hsa-miR-21-5p	RMND5A	required for meiotic nuclear division 5 homolog A
74	89	hsa-miR-21-5p	GRAMD2B	GRAM domain containing 2B
75	89	hsa-miR-21-5p	C7	complement C7
76	89	hsa-miR-21-5p	ALX4	ALX homeobox 4
77	89	hsa-miR-21-5p	STAG2	stromal antigen 2
78	88	hsa-miR-21-5p	ARHGAP24	Rho GTPase activating protein 24
79	88	hsa-miR-21-5p	GLIS2	GLIS family zinc finger 2
80	88	hsa-miR-21-5p	ANKS1B	ankyrin repeat and sterile alpha motif domain containing 1B
81	88	hsa-miR-21-5p	SOX5	SRY-box 5
82	88	hsa-miR-21-5p	NIPAL1	NIPA like domain containing 1
83	88	hsa-miR-21-5p	TMEM170A	transmembrane protein 170A
84	88	hsa-miR-21-5p	RNF103	ring finger protein 103
85	88	hsa-miR-21-5p	LTV1	LTV1 ribosome biogenesis factor
86	88	hsa-miR-21-5p	NEGR1	neuronal growth regulator 1
87	87	hsa-miR-21-5p	CLDN8	claudin 8
88	87	hsa-miR-21-5p	ZNF704	zinc finger protein 704
89	87	hsa-miR-21-5p	HIPK3	homeodomain interacting protein kinase 3
90	87	hsa-miR-21-5p	PPP1R3D	protein phosphatase 1 regulatory subunit 3D
91	87	hsa-miR-21-5p	EPHA4	EPH receptor A4
92	87	hsa-miR-21-5p	ELF2	E74 like ETS transcription factor 2
93	87	hsa-miR-21-5p	RAD51AP1	RAD51 associated protein 1
94	86	hsa-miR-21-5p	MATN2	matrilin 2
95	86	hsa-miR-21-5p	NPPB	natriuretic peptide B
96	86	hsa-miR-21-5p	EHD1	EH domain containing 1
97	86	hsa-miR-21-5p	MCMDC2	minichromosome maintenance domain containing 2
98	86	hsa-miR-21-5p	ITCH	itchy E3 ubiquitin protein ligase
99	86	hsa-miR-21-5p	ATXN10	ataxin 10
100	85	hsa-miR-21-5p	WWP1	WW domain containing E3 ubiquitin protein ligase 1
101	85	hsa-miR-21-5p	NIPAL2	NIPA like domain containing 2
102	85	hsa-miR-21-5p	OLFM3	olfactomedin 3
103	85	hsa-miR-21-5p	MAST4	microtubule associated serine/threonine kinase family member 4
104	85	hsa-miR-21-5p	KCNJ10	potassium voltage-gated channel subfamily J member 10

105	85	hsa-miR-21-5p	TPRG1L	tumor protein p63 regulated 1 like
106	85	hsa-miR-21-5p	SMARCD1	SWI/SNF related, matrix associated, actin dependent regulator of chromatin, subfamily d, member 1
107	85	hsa-miR-21-5p	USP15	ubiquitin specific peptidase 15
108	85	hsa-miR-21-5p	PCSK6	proprotein convertase subtilisin/kexin type 6
109	85	hsa-miR-21-5p	CLIC2	chloride intracellular channel 2
110	85	hsa-miR-21-5p	RASA2	RAS p21 protein activator 2
111	84	hsa-miR-21-5p	ARL1	ADP ribosylation factor like GTPase 1
112	84	hsa-miR-21-5p	LPA	lipoprotein(a)
113	84	hsa-miR-21-5p	GABRB2	gamma-aminobutyric acid type A receptor beta2 subunit
114	83	hsa-miR-21-5p	CUX1	cut like homeobox 1
115	83	hsa-miR-21-5p	RASGRP1	RAS guanyl releasing protein 1
116	83	hsa-miR-21-5p	MINDY2	MINDY lysine 48 deubiquitinase 2
117	83	hsa-miR-21-5p	CHIC1	cysteine rich hydrophobic domain 1
118	83	hsa-miR-21-5p	FDX1	ferredoxin 1
119	83	hsa-miR-21-5p	NFIB	nuclear factor I B
120	83	hsa-miR-21-5p	STK40	serine/threonine kinase 40
121	83	hsa-miR-21-5p	UBR3	ubiquitin protein ligase E3 component n-recognin 3
122	83	hsa-miR-21-5p	LATS1	large tumor suppressor kinase 1
123	83	hsa-miR-21-5p	CSRNP3	cysteine and serine rich nuclear protein 3
124	82	hsa-miR-21-5p	RP2	RP2, ARL3 GTPase activating protein
125	82	hsa-miR-21-5p	SMAD7	SMAD family member 7
126	82	hsa-miR-21-5p	PLAA	phospholipase A2 activating protein
127	82	hsa-miR-21-5p	BAHD1	bromo adjacent homology domain containing 1
128	82	hsa-miR-21-5p	SPEF2	sperm flagellar 2
129	82	hsa-miR-21-5p	MED21	mediator complex subunit 21
130	82	hsa-miR-21-5p	THRB	thyroid hormone receptor beta
131	82	hsa-miR-21-5p	PITX2	paired like homeodomain 2
132	82	hsa-miR-21-5p	BCL11A	BCL11A, BAF complex component
133	82	hsa-miR-21-5p	UBE2D1	ubiquitin conjugating enzyme E2 D1
134	82	hsa-miR-21-5p	FRMD3	FERM domain containing 3
135	81	hsa-miR-21-5p	STK38L	serine/threonine kinase 38 like
136	81	hsa-miR-21-5p	BEST3	bestrophin 3
137	81	hsa-miR-21-5p	ZDHHC17	zinc finger DHHC-type containing 17
138	81	hsa-miR-21-5p	RBMS3	RNA binding motif single stranded interacting protein 3
139	81	hsa-miR-21-5p	DLGAP1	DLG associated protein 1
140	81	hsa-miR-21-5p	KHDC1L	KH domain containing 1 like
141	81	hsa-miR-21-5p	TENT5A	terminal nucleotidyltransferase 5A
142	81	hsa-miR-21-5p	CDH7	cadherin 7
143	81	hsa-miR-21-5p	KHDC1	KH domain containing 1
144	80	hsa-miR-21-5p	SOS2	SOS Ras/Rho guanine nucleotide exchange factor 2
145	80	hsa-miR-21-5p	KLF6	Kruppel like factor 6
146	80	hsa-miR-21-5p	KBTBD6	kelch repeat and BTB domain containing 6
147	80	hsa-miR-21-5p	MSH2	mutS homolog 2
148	80	hsa-miR-21-5p	PTPN20	protein tyrosine phosphatase, non-receptor type 20
149	80	hsa-miR-21-5p	DUSP8	dual specificity phosphatase 8
150	80	hsa-miR-21-5p	CNOT6	CCR4-NOT transcription complex subunit 6
151	79	hsa-miR-21-5p	EDIL3	EGF like repeats and discoidin domains 3
152	79	hsa-miR-21-5p	FANCC	FA complementation group C
153	79	hsa-miR-21-5p	IL6R	interleukin 6 receptor
154	79	hsa-miR-21-5p	ING3	inhibitor of growth family member 3
155	79	hsa-miR-21-5p	SETD9	SET domain containing 9
156	79	hsa-miR-21-5p	BBS12	Bardet-Biedl syndrome 12
157	79	hsa-miR-21-5p	ATP2B4	ATPase plasma membrane Ca2+ transporting 4

158	78	hsa-miR-21-5p	MTMR12	myotubularin related protein 12
159	78	hsa-miR-21-5p	PRKCE	protein kinase C epsilon
160	78	hsa-miR-21-5p	44084	septin 10
161	78	hsa-miR-21-5p	RAB11A	RAB11A, member RAS oncogene family
162	78	hsa-miR-21-5p	FBXO11	F-box protein 11
163	78	hsa-miR-21-5p	GNAQ	G protein subunit alpha q
164	78	hsa-miR-21-5p	KIF6	kinesin family member 6
165	78	hsa-miR-21-5p	ZSWIM6	zinc finger SWIM-type containing 6
166	78	hsa-miR-21-5p	FKBP14	FKBP prolyl isomerase 14
167	78	hsa-miR-21-5p	FBXO28	F-box protein 28
168	78	hsa-miR-21-5p	CNBP	CCHC-type zinc finger nucleic acid binding protein
169	78	hsa-miR-21-5p	PCBP1	poly(rC) binding protein 1
170	78	hsa-miR-21-5p	XKR6	XK related 6
171	77	hsa-miR-21-5p	SAMD8	sterile alpha motif domain containing 8
172	77	hsa-miR-21-5p	IRAK1BP1	interleukin 1 receptor associated kinase 1 binding protein 1
173	77	hsa-miR-21-5p	N4BP2L1	NEDD4 binding protein 2 like 1
174	77	hsa-miR-21-5p	ALDH1A1	aldehyde dehydrogenase 1 family member A1
175	77	hsa-miR-21-5p	SLC7A6	solute carrier family 7 member 6
176	77	hsa-miR-21-5p	MAP2K3	mitogen-activated protein kinase kinase 3
177	77	hsa-miR-21-5p	HSD17B7	hydroxysteroid 17-beta dehydrogenase 7
178	76	hsa-miR-21-5p	VSNL1	visinin like 1
179	76	hsa-miR-21-5p	INPP4A	inositol polyphosphate-4-phosphatase type I A
180	76	hsa-miR-21-5p	ATRNL1	attractin like 1
181	76	hsa-miR-21-5p	ACVR1C	activin A receptor type 1C
182	76	hsa-miR-21-5p	MIA3	MIA SH3 domain ER export factor 3
183	76	hsa-miR-21-5p	SATB1	SATB homeobox 1
184	76	hsa-miR-21-5p	PDAP1	PDGFA associated protein 1
185	76	hsa-miR-21-5p	DCAF7	DDB1 and CUL4 associated factor 7
186	76	hsa-miR-21-5p	SRL	sarcalumenin
187	76	hsa-miR-21-5p	MBLAC2	metallo-beta-lactamase domain containing 2
188	76	hsa-miR-21-5p	NCAPG	non-SMC condensin I complex subunit G
189	76	hsa-miR-21-5p	SLC9A6	solute carrier family 9 member A6
190	76	hsa-miR-21-5p	TNRC6B	trinucleotide repeat containing 6B
191	76	hsa-miR-21-5p	CLIP4	CAP-Gly domain containing linker protein family member 4
192	76	hsa-miR-21-5p	ST6GAL1	ST6 beta-galactoside alpha-2,6-sialyltransferase 1
193	76	hsa-miR-21-5p	CFL2	cofilin 2
194	75	hsa-miR-21-5p	ESYT2	extended synaptotagmin 2
195	75	hsa-miR-21-5p	VASH2	vasohibin 2
196	75	hsa-miR-21-5p	AGO4	argonaute RISC catalytic component 4
197	75	hsa-miR-21-5p	RTKN2	rhotekin 2
198	75	hsa-miR-21-5p	C17orf75	chromosome 17 open reading frame 75
199	75	hsa-miR-21-5p	ZCCHC3	zinc finger CCHC-type containing 3
200	75	hsa-miR-21-5p	PRRC1	proline rich coiled-coil 1
201	74	hsa-miR-21-5p	CDC25A	cell division cycle 25A
202	74	hsa-miR-21-5p	G2E3	G2/M-phase specific E3 ubiquitin protein ligase
203	74	hsa-miR-21-5p	CBX4	chromobox 4
204	74	hsa-miR-21-5p	SOCS6	suppressor of cytokine signaling 6
205	74	hsa-miR-21-5p	MTPN	myotrophin
206	73	hsa-miR-21-5p	KLF12	Kruppel like factor 12
207	73	hsa-miR-21-5p	POLR3B	RNA polymerase III subunit B
208	73	hsa-miR-21-5p	TAF5	TATA-box binding protein associated factor 5
209	73	hsa-miR-21-5p	HNRNPU	heterogeneous nuclear ribonucleoprotein U
210	73	hsa-miR-21-5p	ARMCX1	armadillo repeat containing X-linked 1
211	73	hsa-miR-21-5p	LIFR	LIF receptor alpha

212	73	hsa-miR-21-5p	PPARA	peroxisome proliferator activated receptor alpha
213	73	hsa-miR-21-5p	UNC80	unc-80 homolog, NALCN channel complex subunit
214	73	hsa-miR-21-5p	ZNF99	zinc finger protein 99
215	73	hsa-miR-21-5p	SC5D	sterol-C5-desaturase
216	73	hsa-miR-21-5p	NCOA2	nuclear receptor coactivator 2
217	73	hsa-miR-21-5p	GLDN	gliomedin
218	72	hsa-miR-21-5p	ZFP36L2	ZFP36 ring finger protein like 2
219	72	hsa-miR-21-5p	LYRM7	LYR motif containing 7
220	72	hsa-miR-21-5p	IST1	IST1, ESCRT-III associated factor
221	72	hsa-miR-21-5p	KIN	Kin17 DNA and RNA binding protein
222	72	hsa-miR-21-5p	THPO	thrombopoietin
223	72	hsa-miR-21-5p	NFAT5	nuclear factor of activated T cells 5
224	72	hsa-miR-21-5p	PIK3R1	phosphoinositide-3-kinase regulatory subunit 1
225	72	hsa-miR-21-5p	POM121	POM121 transmembrane nucleoporin
226	72	hsa-miR-21-5p	PRPF39	pre-mRNA processing factor 39
227	72	hsa-miR-21-5p	ADAMTS3	ADAM metallopeptidase with thrombospondin type 1 motif 3
228	72	hsa-miR-21-5p	NEK11	NIMA related kinase 11
229	72	hsa-miR-21-5p	PHF6	PHD finger protein 6
230	72	hsa-miR-21-5p	TMEM163	transmembrane protein 163
231	71	hsa-miR-21-5p	MBNL1	muscleblind like splicing regulator 1
232	71	hsa-miR-21-5p	JPH1	junctophilin 1
233	71	hsa-miR-21-5p	ST3GAL6	ST3 beta-galactoside alpha-2,3-sialyltransferase 6
234	71	hsa-miR-21-5p	B3GAT2	beta-1,3-glucuronyltransferase 2
235	71	hsa-miR-21-5p	EFNA1	ephrin A1
236	71	hsa-miR-21-5p	SLC16A10	solute carrier family 16 member 10
237	71	hsa-miR-21-5p	LAMA4	laminin subunit alpha 4
238	71	hsa-miR-21-5p	RTN4	reticulon 4
239	71	hsa-miR-21-5p	FGD4	FYVE, RhoGEF and PH domain containing 4
240	71	hsa-miR-21-5p	BMPR2	bone morphogenetic protein receptor type 2
241	71	hsa-miR-21-5p	S100A10	S100 calcium binding protein A10
242	71	hsa-miR-21-5p	PHF14	PHD finger protein 14
243	71	hsa-miR-21-5p	PGM1	phosphoglucomutase 1
244	70	hsa-miR-21-5p	TNPO1	transportin 1
245	70	hsa-miR-21-5p	DEPDC4	DEP domain containing 4
246	70	hsa-miR-21-5p	CALCB	calcitonin related polypeptide beta
247	70	hsa-miR-21-5p	MATR3	matrin 3
248	70	hsa-miR-21-5p	PGRMC2	progesterone receptor membrane component 2
249	70	hsa-miR-21-5p	PREX2	phosphatidylinositol-3,4,5-trisphosphate dependent Rac exchange factor 2
250	70	hsa-miR-21-5p	PM20D2	peptidase M20 domain containing 2
251	70	hsa-miR-21-5p	PIKFYVE	phosphoinositide kinase, FYVE-type zinc finger containing
252	70	hsa-miR-21-5p	CCDC121	coiled-coil domain containing 121
253	70	hsa-miR-21-5p	ZNF592	zinc finger protein 592
254	70	hsa-miR-21-5p	GAB1	GRB2 associated binding protein 1
255	70	hsa-miR-21-5p	TESK2	testis associated actin remodelling kinase 2
256	69	hsa-miR-21-5p	GTPBP10	GTP binding protein 10
257	69	hsa-miR-21-5p	SP100	SP100 nuclear antigen
258	69	hsa-miR-21-5p	SEZ6L	seizure related 6 homolog like
259	69	hsa-miR-21-5p	SLC17A5	solute carrier family 17 member 5
260	68	hsa-miR-21-5p	INTS6	integrator complex subunit 6
261	68	hsa-miR-21-5p	GIMAP5	GTPase, IMAP family member 5
262	68	hsa-miR-21-5p	GIMAP1-GIMAP5	GIMAP1-GIMAP5 readthrough
263	68	hsa-miR-21-5p	TBL1XR1	transducin beta like 1 X-linked receptor 1

264	68	hsa-miR-21-5p	SUZ12	SUZ12, polycomb repressive complex 2 subunit
265	68	hsa-miR-21-5p	ARHGAP32	Rho GTPase activating protein 32
266	68	hsa-miR-21-5p	CTR9	CTR9 homolog, Paf1/RNA polymerase II complex component
267	67	hsa-miR-21-5p	CPNE4	copine 4
268	67	hsa-miR-21-5p	CRYBG2	crystallin beta-gamma domain containing 2
269	67	hsa-miR-21-5p	HIC2	HIC ZBTB transcriptional repressor 2
270	67	hsa-miR-21-5p	CEP97	centrosomal protein 97
271	67	hsa-miR-21-5p	ADNP	activity dependent neuroprotector homeobox
272	67	hsa-miR-21-5p	MSX1	msh homeobox 1
273	67	hsa-miR-21-5p	PIGG	phosphatidylinositol glycan anchor biosynthesis class G
274	67	hsa-miR-21-5p	SLC22A15	solute carrier family 22 member 15
275	67	hsa-miR-21-5p	SOX7	SRY-box 7
276	67	hsa-miR-21-5p	TET1	tet methylcytosine dioxygenase 1
277	67	hsa-miR-21-5p	ZNF200	zinc finger protein 200
278	66	hsa-miR-21-5p	DAG1	dystroglycan 1
279	66	hsa-miR-21-5p	MEF2C	myocyte enhancer factor 2C
280	66	hsa-miR-21-5p	ITGB8	integrin subunit beta 8
281	66	hsa-miR-21-5p	TAGAP	T cell activation RhoGTPase activating protein
282	66	hsa-miR-21-5p	ST8SIA3	ST8 alpha-N-acetyl-neuraminide alpha-2,8-sialyltransferase 3
283	66	hsa-miR-21-5p	CLTRN	collectrin, amino acid transport regulator
284	66	hsa-miR-21-5p	ALX1	ALX homeobox 1
285	66	hsa-miR-21-5p	PLP1	proteolipid protein 1
286	66	hsa-miR-21-5p	GPC4	glypican 4
287	65	hsa-miR-21-5p	RNF2	ring finger protein 2
288	65	hsa-miR-21-5p	RASAL2	RAS protein activator like 2
289	65	hsa-miR-21-5p	NSL1	NSL1, MIS12 kinetochore complex component
290	65	hsa-miR-21-5p	AIF1L	allograft inflammatory factor 1 like
291	65	hsa-miR-21-5p	RAPGEF6	Rap guanine nucleotide exchange factor 6
292	65	hsa-miR-21-5p	PRMT9	protein arginine methyltransferase 9
293	65	hsa-miR-21-5p	LUM	lumican
294	65	hsa-miR-21-5p	SLC35F5	solute carrier family 35 member F5
295	65	hsa-miR-21-5p	ZNF728	zinc finger protein 728
296	65	hsa-miR-21-5p	FOXO3	forkhead box O3
297	65	hsa-miR-21-5p	BMP3	bone morphogenetic protein 3
298	65	hsa-miR-21-5p	MKNK2	MAP kinase interacting serine/threonine kinase 2
299	64	hsa-miR-21-5p	ZGRF1	zinc finger GRF-type containing 1
300	64	hsa-miR-21-5p	FGF1	fibroblast growth factor 1
301	64	hsa-miR-21-5p	TBX2	T-box 2
302	64	hsa-miR-21-5p	SCRN1	secernin 1
303	64	hsa-miR-21-5p	CD69	CD69 molecule
304	64	hsa-miR-21-5p	NRXN3	neurexin 3
305	64	hsa-miR-21-5p	GP9	glycoprotein IX platelet
306	64	hsa-miR-21-5p	FMN1	formin 1
307	64	hsa-miR-21-5p	ASF1A	anti-silencing function 1A histone chaperone
308	63	hsa-miR-21-5p	HAX1	HCLS1 associated protein X-1
309	63	hsa-miR-21-5p	TRIP11	thyroid hormone receptor interactor 11
310	63	hsa-miR-21-5p	BNC2	basonuclin 2
311	63	hsa-miR-21-5p	SOWAHC	sosondowah ankyrin repeat domain family member C
312	63	hsa-miR-21-5p	RETSAT	retinol saturase
313	63	hsa-miR-21-5p	SLC5A7	solute carrier family 5 member 7
314	63	hsa-miR-21-5p	SPDYA	speedy/RINGO cell cycle regulator family member A
315	63	hsa-miR-21-5p	PTPN4	protein tyrosine phosphatase, non-receptor type 4
316	62	hsa-miR-21-5p	LANCL3	LanC like 3
317	62	hsa-miR-21-5p	PRICKLE2	prickle planar cell polarity protein 2

318	62	hsa-miR-21-5p	CATSPERE	catsper channel auxiliary subunit epsilon
319	62	hsa-miR-21-5p	OLR1	oxidized low density lipoprotein receptor 1
320	62	hsa-miR-21-5p	BBIP1	BBSome interacting protein 1
321	62	hsa-miR-21-5p	PCBP2	poly(rC) binding protein 2
322	62	hsa-miR-21-5p	LZTFL1	leucine zipper transcription factor like 1
323	62	hsa-miR-21-5p	ANXA1	annexin A1
324	61	hsa-miR-21-5p	KLHL42	kelch like family member 42
325	61	hsa-miR-21-5p	TRAPPC8	trafficking protein particle complex 8
326	61	hsa-miR-21-5p	ATP11B	ATPase phospholipid transporting 11B (putative)
327	61	hsa-miR-21-5p	DIPK2A	divergent protein kinase domain 2A
328	61	hsa-miR-21-5p	AKAP6	A-kinase anchoring protein 6
329	61	hsa-miR-21-5p	ROBO2	roundabout guidance receptor 2
330	61	hsa-miR-21-5p	MAP10	microtubule associated protein 10
331	61	hsa-miR-21-5p	EPM2AIP1	EPM2A interacting protein 1
332	61	hsa-miR-21-5p	SESN1	sestrin 1
333	61	hsa-miR-21-5p	SLC2A4RG	SLC2A4 regulator
334	61	hsa-miR-21-5p	GCLM	glutamate-cysteine ligase modifier subunit
335	61	hsa-miR-21-5p	ACVR2A	activin A receptor type 2A
336	61	hsa-miR-21-5p	LEMD3	LEM domain containing 3
337	61	hsa-miR-21-5p	SGO1	shugoshin 1
338	60	hsa-miR-21-5p	RASEF	RAS and EF-hand domain containing
339	60	hsa-miR-21-5p	APAF1	apoptotic peptidase activating factor 1
340	60	hsa-miR-21-5p	GRM1	glutamate metabotropic receptor 1
341	60	hsa-miR-21-5p	TGFB2	transforming growth factor beta 2
342	60	hsa-miR-21-5p	GAPT	GRB2 binding adaptor protein, transmembrane
343	60	hsa-miR-21-5p	TFDP1	transcription factor Dp-1
344	59	hsa-miR-21-5p	ABCD3	ATP binding cassette subfamily D member 3
345	59	hsa-miR-21-5p	PDLIM5	PDZ and LIM domain 5
346	59	hsa-miR-21-5p	NR2C2	nuclear receptor subfamily 2 group C member 2
347	59	hsa-miR-21-5p	CADM2	cell adhesion molecule 2
348	59	hsa-miR-21-5p	SAMD9	sterile alpha motif domain containing 9
349	59	hsa-miR-21-5p	EXTL2	exostosin like glycosyltransferase 2
350	59	hsa-miR-21-5p	ANKRD46	ankyrin repeat domain 46
351	59	hsa-miR-21-5p	SCN8A	sodium voltage-gated channel alpha subunit 8
352	59	hsa-miR-21-5p	E2F3	E2F transcription factor 3
353	59	hsa-miR-21-5p	AKIRIN1	akirin 1
354	59	hsa-miR-21-5p	PCGF5	polycomb group ring finger 5
355	59	hsa-miR-21-5p	NTRK2	neurotrophic receptor tyrosine kinase 2
356	59	hsa-miR-21-5p	ZNF527	zinc finger protein 527
357	58	hsa-miR-21-5p	ZNF217	zinc finger protein 217
358	58	hsa-miR-21-5p	GPR34	G protein-coupled receptor 34
359	58	hsa-miR-21-5p	ZXDA	zinc finger X-linked duplicated A
360	58	hsa-miR-21-5p	GCNT2	glucosaminyl (N-acetyl) transferase 2 (I blood group)
361	58	hsa-miR-21-5p	LCOR	ligand dependent nuclear receptor corepressor
362	58	hsa-miR-21-5p	MYO5B	myosin VB
363	58	hsa-miR-21-5p	MAN1A2	mannosidase alpha class 1A member 2
364	58	hsa-miR-21-5p	TNFAIP3	TNF alpha induced protein 3
365	58	hsa-miR-21-5p	HGF	hepatocyte growth factor
366	58	hsa-miR-21-5p	ZNF805	zinc finger protein 805
367	58	hsa-miR-21-5p	DLX2	distal-less homeobox 2
368	58	hsa-miR-21-5p	RNF32	ring finger protein 32
369	58	hsa-miR-21-5p	ZNF35	zinc finger protein 35
370	58	hsa-miR-21-5p	MAP3K2	mitogen-activated protein kinase kinase kinase 2
371	58	hsa-miR-21-5p	APH1B	aph-1 homolog B, gamma-secretase subunit

372	58	hsa-miR-21-5p	PID1	phosphotyrosine interaction domain containing 1
373	58	hsa-miR-21-5p	RASSF6	Ras association domain family member 6
374	58	hsa-miR-21-5p	ABCA1	ATP binding cassette subfamily A member 1
375	57	hsa-miR-21-5p	FANCM	FA complementation group M
376	57	hsa-miR-21-5p	PRR5L	proline rich 5 like
377	57	hsa-miR-21-5p	LILRB4	leukocyte immunoglobulin like receptor B4
378	57	hsa-miR-21-5p	FXR1	FMR1 autosomal homolog 1
379	57	hsa-miR-21-5p	TLR4	toll like receptor 4
380	57	hsa-miR-21-5p	KCTD18	potassium channel tetramerization domain containing 18
381	57	hsa-miR-21-5p	PTPRG	protein tyrosine phosphatase, receptor type G
382	57	hsa-miR-21-5p	WWC2	WW and C2 domain containing 2
383	57	hsa-miR-21-5p	FASTKD2	FAST kinase domains 2
384	57	hsa-miR-21-5p	COX15	cytochrome c oxidase assembly homolog COX15
385	57	hsa-miR-21-5p	FRS2	fibroblast growth factor receptor substrate 2
386	57	hsa-miR-21-5p	COL4A1	collagen type IV alpha 1 chain
387	57	hsa-miR-21-5p	FAM177B	family with sequence similarity 177 member B
388	57	hsa-miR-21-5p	CD1A	CD1a molecule
389	56	hsa-miR-21-5p	FBXO46	F-box protein 46
390	56	hsa-miR-21-5p	SOCS5	suppressor of cytokine signaling 5
391	56	hsa-miR-21-5p	SPON1	spondin 1
392	56	hsa-miR-21-5p	SGK3	serum/glucocorticoid regulated kinase family member 3
393	56	hsa-miR-21-5p	ARHGEF7	Rho guanine nucleotide exchange factor 7
394	56	hsa-miR-21-5p	TAT	tyrosine aminotransferase
395	56	hsa-miR-21-5p	SPAG11B	sperm associated antigen 11B
396	56	hsa-miR-21-5p	C8orf44-SGK3	C8orf44-SGK3 readthrough
397	55	hsa-miR-21-5p	ULBP3	UL16 binding protein 3
398	55	hsa-miR-21-5p	SLC8A3	solute carrier family 8 member A3
399	55	hsa-miR-21-5p	MXD3	MAX dimerization protein 3
400	55	hsa-miR-21-5p	GALNT12	polypeptide N-acetylgalactosaminyltransferase 12
401	55	hsa-miR-21-5p	CENPQ	centromere protein Q
402	55	hsa-miR-21-5p	ATF7IP	activating transcription factor 7 interacting protein
403	55	hsa-miR-21-5p	MAPRE1	microtubule associated protein RP/EB family member 1
404	55	hsa-miR-21-5p	AMER1	APC membrane recruitment protein 1
405	55	hsa-miR-21-5p	HNMT	histamine N-methyltransferase
406	55	hsa-miR-21-5p	CALN1	calneuron 1
407	55	hsa-miR-21-5p	ZNF576	zinc finger protein 576
408	55	hsa-miR-21-5p	IPO11	importin 11
409	55	hsa-miR-21-5p	RASSF8	Ras association domain family member 8
410	54	hsa-miR-21-5p	PRPF4B	pre-mRNA processing factor 4B
411	54	hsa-miR-21-5p	EML6	EMAP like 6
412	54	hsa-miR-21-5p	NAA50	N(alpha)-acetyltransferase 50, NatE catalytic subunit
413	54	hsa-miR-21-5p	RBM17	RNA binding motif protein 17
414	54	hsa-miR-21-5p	EIF2S1	eukaryotic translation initiation factor 2 subunit alpha
415	54	hsa-miR-21-5p	CERS5	ceramide synthase 5
416	54	hsa-miR-21-5p	DSC2	desmocollin 2
417	54	hsa-miR-21-5p	CRTAM	cytotoxic and regulatory T cell molecule
418	54	hsa-miR-21-5p	FAM20B	FAM20B, glycosaminoglycan xylosylkinase
419	54	hsa-miR-21-5p	SMURF2	SMAD specific E3 ubiquitin protein ligase 2
420	54	hsa-miR-21-5p	DDAH1	dimethylarginine dimethylaminohydrolase 1
421	54	hsa-miR-21-5p	TC2N	tandem C2 domains, nuclear
422	53	hsa-miR-21-5p	IQCH	IQ motif containing H
423	53	hsa-miR-21-5p	TMED10	transmembrane p24 trafficking protein 10
424	53	hsa-miR-21-5p	CDK2AP1	cyclin dependent kinase 2 associated protein 1
425	53	hsa-miR-21-5p	LANCL1	LanC like 1

426	53	hsa-miR-21-5p	ANKRD37	ankyrin repeat domain 37
427	53	hsa-miR-21-5p	DIRAS2	DIRAS family GTPase 2
428	53	hsa-miR-21-5p	LGR6	leucine rich repeat containing G protein-coupled receptor 6
429	53	hsa-miR-21-5p	REPS1	RALBP1 associated Eps domain containing 1
430	53	hsa-miR-21-5p	STRN	striatin
431	53	hsa-miR-21-5p	LCORL	ligand dependent nuclear receptor corepressor like
432	53	hsa-miR-21-5p	ZNF680	zinc finger protein 680
433	53	hsa-miR-21-5p	ITIH5	inter-alpha-trypsin inhibitor heavy chain family member 5
434	53	hsa-miR-21-5p	PURB	purine rich element binding protein B
435	53	hsa-miR-21-5p	PDZD8	PDZ domain containing 8
436	53	hsa-miR-21-5p	RIPOR2	RHO family interacting cell polarization regulator 2
437	52	hsa-miR-21-5p	CETN1	centrin 1
438	52	hsa-miR-21-5p	CASTOR1	cytosolic arginine sensor for mTORC1 subunit 1
439	52	hsa-miR-21-5p	DDX55	DEAD-box helicase 55
440	52	hsa-miR-21-5p	KIAA0825	KIAA0825
441	52	hsa-miR-21-5p	IGF2BP3	insulin like growth factor 2 mRNA binding protein 3
442	52	hsa-miR-21-5p	DNM1L	dynamin 1 like
443	52	hsa-miR-21-5p	CTSC	cathepsin C
444	52	hsa-miR-21-5p	STK3	serine/threonine kinase 3
445	52	hsa-miR-21-5p	LIX1L	limb and CNS expressed 1 like
446	52	hsa-miR-21-5p	KLF5	Kruppel like factor 5
447	51	hsa-miR-21-5p	SUV39H2	suppressor of variegation 3-9 homolog 2
448	51	hsa-miR-21-5p	C5orf58	chromosome 5 open reading frame 58
449	51	hsa-miR-21-5p	PAG1	phosphoprotein membrane anchor with glycosphingolipid microdomains 1
450	51	hsa-miR-21-5p	DENND1B	DENN domain containing 1B
451	51	hsa-miR-21-5p	CLIC5	chloride intracellular channel 5
452	51	hsa-miR-21-5p	PAIP2B	poly(A) binding protein interacting protein 2B
453	51	hsa-miR-21-5p	ARFRP1	ADP ribosylation factor related protein 1
454	51	hsa-miR-21-5p	LRRIQ3	leucine rich repeats and IQ motif containing 3
455	51	hsa-miR-21-5p	DNAJB9	DnaJ heat shock protein family (Hsp40) member B9
456	51	hsa-miR-21-5p	BRCC3	BRCA1/BRCA2-containing complex subunit 3
457	50	hsa-miR-21-5p	NOP14	NOP14 nucleolar protein
458	50	hsa-miR-21-5p	ZNF326	zinc finger protein 326
459	50	hsa-miR-21-5p	PDHA1	pyruvate dehydrogenase E1 alpha 1 subunit
460	50	hsa-miR-21-5p	SACM1L	SAC1 like phosphatidylinositide phosphatase
461	50	hsa-miR-21-5p	AMBN	ameloblastin
462	50	hsa-miR-21-5p	CA1	carbonic anhydrase 1
463	50	hsa-miR-21-5p	HNRNPK	heterogeneous nuclear ribonucleoprotein K
464	50	hsa-miR-21-5p	GPRASP2	G protein-coupled receptor associated sorting protein 2
465	50	hsa-miR-21-5p	ARMCX5-GPRASP2	ARMCX5-GPRASP2 readthrough
466	50	hsa-miR-21-5p	RNF180	ring finger protein 180
467	50	hsa-miR-21-5p	TMEM246	transmembrane protein 246
468	50	hsa-miR-21-5p	ADGRE5	adhesion G protein-coupled receptor E5
469	50	hsa-miR-21-5p	ELOVL7	ELOVL fatty acid elongase 7

Table 3. List of all hsa-miR-21-3p targets. Identification of all the predicted targets of hsa-miR-21-3p using miRDB.

Target Rank	Target Score	miRNA Name	Gene Symbol	Gene Description
1	99	hsa-miR-21-3p	STK38L	serine/threonine kinase 38 like
2	98	hsa-miR-21-3p	PCDH19	protocadherin 19
3	96	hsa-miR-21-3p	LAMP1	lysosomal associated membrane protein 1
4	96	hsa-miR-21-3p	GRIA2	glutamate ionotropic receptor AMPA type subunit 2
5	96	hsa-miR-21-3p	TOGARAM1	TOG array regulator of axonemal microtubules 1
6	96	hsa-miR-21-3p	ATP1B1	ATPase Na+/K+ transporting subunit beta 1
7	96	hsa-miR-21-3p	TSC22D2	TSC22 domain family member 2
8	96	hsa-miR-21-3p	NAP1L5	nucleosome assembly protein 1 like 5
9	95	hsa-miR-21-3p	UBE4B	ubiquitination factor E4B
10	95	hsa-miR-21-3p	ZNF326	zinc finger protein 326
11	95	hsa-miR-21-3p	CDK8	cyclin dependent kinase 8
12	94	hsa-miR-21-3p	MAP2K4	mitogen-activated protein kinase kinase 4
13	94	hsa-miR-21-3p	AKAP11	A-kinase anchoring protein 11
14	94	hsa-miR-21-3p	GPM6A	glycoprotein M6A
15	94	hsa-miR-21-3p	MAP3K1	mitogen-activated protein kinase kinase kinase 1
16	94	hsa-miR-21-3p	PARD3B	par-3 family cell polarity regulator beta
17	94	hsa-miR-21-3p	ALCAM	activated leukocyte cell adhesion molecule
18	94	hsa-miR-21-3p	FOXO3	forkhead box O3
19	94	hsa-miR-21-3p	FYTTD1	forty-two-three domain containing 1
20	94	hsa-miR-21-3p	PHYHIPL	phytanoyl-CoA 2-hydroxylase interacting protein like
21	93	hsa-miR-21-3p	ELL2	elongation factor for RNA polymerase II 2
22	93	hsa-miR-21-3p	RRAGB	Ras related GTP binding B
23	93	hsa-miR-21-3p	ZIC5	Zic family member 5
24	93	hsa-miR-21-3p	SERINC5	serine incorporator 5
25	92	hsa-miR-21-3p	SMAD7	SMAD family member 7
26	92	hsa-miR-21-3p	CAMSAP2	calmodulin regulated spectrin associated protein family member 2
27	92	hsa-miR-21-3p	OR10W1	olfactory receptor family 10 subfamily W member 1
28	92	hsa-miR-21-3p	CDK14	cyclin dependent kinase 14
29	91	hsa-miR-21-3p	SEH1L	SEH1 like nucleoporin
30	91	hsa-miR-21-3p	CPEB2	cytoplasmic polyadenylation element binding protein 2
31	91	hsa-miR-21-3p	TIAM1	T cell lymphoma invasion and metastasis 1
32	91	hsa-miR-21-3p	MAGEB18	MAGE family member B18
33	91	hsa-miR-21-3p	DDX4	DEAD-box helicase 4
34	91	hsa-miR-21-3p	SNRK	SNF related kinase
35	91	hsa-miR-21-3p	HS2ST1	heparan sulfate 2-O-sulfotransferase 1
36	91	hsa-miR-21-3p	PPP1R2	protein phosphatase 1 regulatory inhibitor subunit 2
37	91	hsa-miR-21-3p	GAD2	glutamate decarboxylase 2
38	91	hsa-miR-21-3p	TMEFF1	transmembrane protein with EGF like and two follistatin like domains 1
39	91	hsa-miR-21-3p	TMEM164	transmembrane protein 164
40	90	hsa-miR-21-3p	UMAD1	UBAP1-MVB12-associated (UMA) domain containing 1
41	90	hsa-miR-21-3p	RC3H1	ring finger and CCCH-type domains 1
42	90	hsa-miR-21-3p	SRSF2	serine and arginine rich splicing factor 2
43	90	hsa-miR-21-3p	ZC3H12C	zinc finger CCCH-type containing 12C
44	90	hsa-miR-21-3p	BCL2L11	BCL2 like 11
45	90	hsa-miR-21-3p	FAM83H	family with sequence similarity 83 member H
46	90	hsa-miR-21-3p	RTN3	reticulon 3
47	89	hsa-miR-21-3p	HSD17B11	hydroxysteroid 17-beta dehydrogenase 11
48	89	hsa-miR-21-3p	RIPOR3	RIPOR family member 3
49	89	hsa-miR-21-3p	FSBP	fibrinogen silencer binding protein
50	89	hsa-miR-21-3p	WDR33	WD repeat domain 33

51	89	hsa-miR-21-3p	TAP1	transporter 1, ATP binding cassette subfamily B member
52	89	hsa-miR-21-3p	RDX	radixin
53	89	hsa-miR-21-3p	USP51	ubiquitin specific peptidase 51
54	89	hsa-miR-21-3p	FXR1	FMR1 autosomal homolog 1
55	89	hsa-miR-21-3p	PWWP3B	PWWP domain containing 3B
56	89	hsa-miR-21-3p	TANGO2	transport and golgi organization 2 homolog
57	89	hsa-miR-21-3p	SLC35B3	solute carrier family 35 member B3
58	89	hsa-miR-21-3p	RAD54B	RAD54 homolog B
59	89	hsa-miR-21-3p	TOPORS	TOP1 binding arginine/serine rich protein
60	89	hsa-miR-21-3p	FOXR2	forkhead box R2
61	88	hsa-miR-21-3p	PDCD10	programmed cell death 10
62	88	hsa-miR-21-3p	MLX	MLX, MAX dimerization protein
63	88	hsa-miR-21-3p	UBN1	ubinuclein 1
64	88	hsa-miR-21-3p	CDADC1	cytidine and dCMP deaminase domain containing 1
65	88	hsa-miR-21-3p	TFG	TRK-fused gene
66	88	hsa-miR-21-3p	TMA16	translation machinery associated 16 homolog
67	88	hsa-miR-21-3p	SAMD8	sterile alpha motif domain containing 8
68	87	hsa-miR-21-3p	C9orf72	chromosome 9 open reading frame 72
69	87	hsa-miR-21-3p	POLR2M	RNA polymerase II subunit M
70	87	hsa-miR-21-3p	RIPK1	receptor interacting serine/threonine kinase 1
71	87	hsa-miR-21-3p	MYCL	MYCL proto-oncogene, bHLH transcription factor
72	87	hsa-miR-21-3p	TRIO	trio Rho guanine nucleotide exchange factor
73	87	hsa-miR-21-3p	FAM117A	family with sequence similarity 117 member A
74	87	hsa-miR-21-3p	ZNF800	zinc finger protein 800
75	87	hsa-miR-21-3p	CCDC85A	coiled-coil domain containing 85A
76	87	hsa-miR-21-3p	GCOM1	GRINL1A complex locus 1
77	87	hsa-miR-21-3p	WTAP	WT1 associated protein
78	87	hsa-miR-21-3p	GPR161	G protein-coupled receptor 161
79	86	hsa-miR-21-3p	ZFP42	ZFP42 zinc finger protein
80	86	hsa-miR-21-3p	RUNX1T1	RUNX1 translocation partner 1
81	86	hsa-miR-21-3p	PEG3	paternally expressed 3
82	86	hsa-miR-21-3p	AAK1	AP2 associated kinase 1
83	86	hsa-miR-21-3p	CTDSPL	CTD small phosphatase like
84	86	hsa-miR-21-3p	APPL1	adaptor protein, phosphotyrosine interacting with PH domain and leucine zipper 1
85	86	hsa-miR-21-3p	CCL28	C-C motif chemokine ligand 28
86	86	hsa-miR-21-3p	SS18L1	SS18L1, nBAF chromatin remodeling complex subunit
87	85	hsa-miR-21-3p	YWHAG	tyrosine 3-monooxygenase/tryptophan 5-monooxygenase activation protein gamma
88	85	hsa-miR-21-3p	OARD1	O-acyl-ADP-ribose deacylase 1
89	85	hsa-miR-21-3p	NFYB	nuclear transcription factor Y subunit beta
90	85	hsa-miR-21-3p	KIRREL3	kirre like nephrin family adhesion molecule 3
91	85	hsa-miR-21-3p	COL19A1	collagen type XIX alpha 1 chain
92	85	hsa-miR-21-3p	CYP3A5	cytochrome P450 family 3 subfamily A member 5
93	85	hsa-miR-21-3p	DCUN1D4	defective in cullin neddylation 1 domain containing 4
94	84	hsa-miR-21-3p	RPS6KA6	ribosomal protein S6 kinase A6
95	84	hsa-miR-21-3p	HOXC9	homeobox C9
96	84	hsa-miR-21-3p	RPS10-NUDT3	RPS10-NUDT3 readthrough
97	84	hsa-miR-21-3p	CHIC2	cysteine rich hydrophobic domain 2
98	84	hsa-miR-21-3p	SP1	Sp1 transcription factor
99	84	hsa-miR-21-3p	ZZZ3	zinc finger ZZ-type containing 3
100	84	hsa-miR-21-3p	NUDT3	nudix hydrolase 3
101	84	hsa-miR-21-3p	STAU2	staufen double-stranded RNA binding protein 2
102	83	hsa-miR-21-3p	PDIA4	protein disulfide isomerase family A member 4

103	83	hsa-miR-21-3p	PRKG1	protein kinase cGMP-dependent 1
104	83	hsa-miR-21-3p	STMN4	stathmin 4
105	83	hsa-miR-21-3p	PSEN1	presenilin 1
106	83	hsa-miR-21-3p	WDR82	WD repeat domain 82
107	83	hsa-miR-21-3p	C2CD3	C2 calcium dependent domain containing 3
108	83	hsa-miR-21-3p	UCHL5	ubiquitin C-terminal hydrolase L5
109	83	hsa-miR-21-3p	PPP1CC	protein phosphatase 1 catalytic subunit gamma
110	83	hsa-miR-21-3p	RINT1	RAD50 interactor 1
111	83	hsa-miR-21-3p	MMD	monocyte to macrophage differentiation associated
112	83	hsa-miR-21-3p	USP4	ubiquitin specific peptidase 4
113	82	hsa-miR-21-3p	TMEM242	transmembrane protein 242
114	82	hsa-miR-21-3p	UBA3	ubiquitin like modifier activating enzyme 3
115	82	hsa-miR-21-3p	ZNF704	zinc finger protein 704
116	82	hsa-miR-21-3p	PLA2G12B	phospholipase A2 group XIIB
117	82	hsa-miR-21-3p	ING3	inhibitor of growth family member 3
118	82	hsa-miR-21-3p	CDK17	cyclin dependent kinase 17
119	82	hsa-miR-21-3p	GRM5	glutamate metabotropic receptor 5
120	82	hsa-miR-21-3p	DHRSX	dehydrogenase/reductase X-linked
121	82	hsa-miR-21-3p	TMEM94	transmembrane protein 94
122	82	hsa-miR-21-3p	LOX	lysyl oxidase
123	82	hsa-miR-21-3p	HERC3	HECT and RLD domain containing E3 ubiquitin protein ligase 3
124	81	hsa-miR-21-3p	TMEM43	transmembrane protein 43
125	81	hsa-miR-21-3p	ANOS1	anosmin 1
126	81	hsa-miR-21-3p	AGTR1	angiotensin II receptor type 1
127	81	hsa-miR-21-3p	DNAJB4	DnaJ heat shock protein family (Hsp40) member B4
128	81	hsa-miR-21-3p	NAV3	neuron navigator 3
129	81	hsa-miR-21-3p	SMARCE1	SWI/SNF related, matrix associated, actin dependent regulator of chromatin, subfamily e, member 1
130	81	hsa-miR-21-3p	SRCIN1	SRC kinase signaling inhibitor 1
131	81	hsa-miR-21-3p	TMLHE	trimethyllysine hydroxylase, epsilon
132	81	hsa-miR-21-3p	MSANTD3-TMEFF1	MSANTD3-TMEFF1 readthrough
133	81	hsa-miR-21-3p	FAM122B	family with sequence similarity 122B
134	81	hsa-miR-21-3p	PDE4B	phosphodiesterase 4B
135	81	hsa-miR-21-3p	CANX	calnexin
136	81	hsa-miR-21-3p	MBNL3	muscleblind like splicing regulator 3
137	80	hsa-miR-21-3p	CLCN4	chloride voltage-gated channel 4
138	80	hsa-miR-21-3p	NLGN4X	neuroligin 4 X-linked
139	80	hsa-miR-21-3p	PLXNA4	plexin A4
140	80	hsa-miR-21-3p	PPP1R1A	protein phosphatase 1 regulatory inhibitor subunit 1A
141	80	hsa-miR-21-3p	RCOR3	REST corepressor 3
142	80	hsa-miR-21-3p	PCLAF	PCNA clamp associated factor
143	80	hsa-miR-21-3p	CTNNA3	catenin alpha 3
144	80	hsa-miR-21-3p	PDZRN4	PDZ domain containing ring finger 4
145	80	hsa-miR-21-3p	SMG1	SMG1, nonsense mediated mRNA decay associated PI3K related kinase
146	80	hsa-miR-21-3p	ASCC3	activating signal cointegrator 1 complex subunit 3
147	80	hsa-miR-21-3p	DR1	down-regulator of transcription 1
148	80	hsa-miR-21-3p	TMCC3	transmembrane and coiled-coil domain family 3
149	80	hsa-miR-21-3p	COBL	cordon-bleu WH2 repeat protein
150	79	hsa-miR-21-3p	TRAPPC4	trafficking protein particle complex 4
151	79	hsa-miR-21-3p	PPP6R3	protein phosphatase 6 regulatory subunit 3
152	79	hsa-miR-21-3p	RABL3	RAB, member of RAS oncogene family like 3
153	79	hsa-miR-21-3p	ZNF608	zinc finger protein 608
154	79	hsa-miR-21-3p	TM4SF20	transmembrane 4 L six family member 20

155	79	hsa-miR-21-3p	BTBD7	BTB domain containing 7
156	79	hsa-miR-21-3p	SLC35G1	solute carrier family 35 member G1
157	79	hsa-miR-21-3p	GPATCH2L	G-patch domain containing 2 like
158	79	hsa-miR-21-3p	FAT3	FAT atypical cadherin 3
159	79	hsa-miR-21-3p	KIN	Kin17 DNA and RNA binding protein
160	79	hsa-miR-21-3p	CAMK2A	calcium/calmodulin dependent protein kinase II alpha
161	79	hsa-miR-21-3p	PHC3	polyhomeotic homolog 3
162	79	hsa-miR-21-3p	DIRAS1	DIRAS family GTPase 1
163	79	hsa-miR-21-3p	STRAP	serine/threonine kinase receptor associated protein
164	79	hsa-miR-21-3p	UBR5	ubiquitin protein ligase E3 component n-recognin 5
165	78	hsa-miR-21-3p	PRKCH	protein kinase C eta
166	78	hsa-miR-21-3p	TDRP	testis development related protein
167	78	hsa-miR-21-3p	ARF3	ADP ribosylation factor 3
168	78	hsa-miR-21-3p	MAGEL2	MAGE family member L2
169	78	hsa-miR-21-3p	KLHL3	kelch like family member 3
170	78	hsa-miR-21-3p	ZBTB18	zinc finger and BTB domain containing 18
171	78	hsa-miR-21-3p	PTPRK	protein tyrosine phosphatase, receptor type K
172	78	hsa-miR-21-3p	FREM3	FRAS1 related extracellular matrix 3
173	78	hsa-miR-21-3p	DAB2IP	DAB2 interacting protein
174	78	hsa-miR-21-3p	TBC1D19	TBC1 domain family member 19
175	78	hsa-miR-21-3p	SHC3	SHC adaptor protein 3
176	78	hsa-miR-21-3p	STX17	syntaxin 17
177	77	hsa-miR-21-3p	ADIPOQ	adiponectin, C1Q and collagen domain containing
178	77	hsa-miR-21-3p	YIPF4	Yip1 domain family member 4
179	77	hsa-miR-21-3p	MYZAP	myocardial zonula adherens protein
180	77	hsa-miR-21-3p	MARF1	meiosis regulator and mRNA stability factor 1
181	77	hsa-miR-21-3p	RAI14	retinoic acid induced 14
182	77	hsa-miR-21-3p	NFATC3	nuclear factor of activated T cells 3
183	77	hsa-miR-21-3p	YAE1	YAE1, ABCE1 maturation factor
184	77	hsa-miR-21-3p	ARHGEF7	Rho guanine nucleotide exchange factor 7
185	77	hsa-miR-21-3p	NFAT5	nuclear factor of activated T cells 5
186	77	hsa-miR-21-3p	MYB	MYB proto-oncogene, transcription factor
187	77	hsa-miR-21-3p	MAP3K20	mitogen-activated protein kinase kinase kinase 20
188	77	hsa-miR-21-3p	ATP2B1	ATPase plasma membrane Ca2+ transporting 1
189	77	hsa-miR-21-3p	NAA15	N(alpha)-acetyltransferase 15, NatA auxiliary subunit
190	76	hsa-miR-21-3p	PCGF5	polycomb group ring finger 5
191	76	hsa-miR-21-3p	OCRL	OCRL, inositol polyphosphate-5-phosphatase
192	76	hsa-miR-21-3p	C2orf69	chromosome 2 open reading frame 69
193	76	hsa-miR-21-3p	USP44	ubiquitin specific peptidase 44
194	76	hsa-miR-21-3p	CCER1	coiled-coil glutamate rich protein 1
195	76	hsa-miR-21-3p	PKP4	plakophilin 4
196	76	hsa-miR-21-3p	CBX4	chromobox 4
197	76	hsa-miR-21-3p	PLPP4	phospholipid phosphatase 4
198	76	hsa-miR-21-3p	SMG7	SMG7, nonsense mediated mRNA decay factor
199	76	hsa-miR-21-3p	E2F7	E2F transcription factor 7
200	76	hsa-miR-21-3p	KLK11	kallikrein related peptidase 11
201	76	hsa-miR-21-3p	CLEC7A	C-type lectin domain containing 7A
202	75	hsa-miR-21-3p	JCAD	junctional cadherin 5 associated
203	75	hsa-miR-21-3p	RYBP	RING1 and YY1 binding protein
204	74	hsa-miR-21-3p	PCDHA2	protocadherin alpha 2
205	74	hsa-miR-21-3p	MED13	mediator complex subunit 13
206	74	hsa-miR-21-3p	PCDHA7	protocadherin alpha 7
207	74	hsa-miR-21-3p	PCDHA4	protocadherin alpha 4
208	74	hsa-miR-21-3p	DLGAP1	DLG associated protein 1

209	74	hsa-miR-21-3p	PCDHA12	protocadherin alpha 12
210	74	hsa-miR-21-3p	PCDHA1	protocadherin alpha 1
211	74	hsa-miR-21-3p	PCDHA9	protocadherin alpha 9
212	74	hsa-miR-21-3p	PCDHA11	protocadherin alpha 11
213	74	hsa-miR-21-3p	PCDHA3	protocadherin alpha 3
214	74	hsa-miR-21-3p	GAP43	growth associated protein 43
215	74	hsa-miR-21-3p	PCDHA6	protocadherin alpha 6
216	74	hsa-miR-21-3p	SHTN1	shootin 1
217	74	hsa-miR-21-3p	SMAD5	SMAD family member 5
218	74	hsa-miR-21-3p	PPFIA2	PTPRF interacting protein alpha 2
219	74	hsa-miR-21-3p	PCDHAC1	protocadherin alpha subfamily C, 1
220	74	hsa-miR-21-3p	REEP1	receptor accessory protein 1
221	74	hsa-miR-21-3p	PCDHA13	protocadherin alpha 13
222	74	hsa-miR-21-3p	PCDHA8	protocadherin alpha 8
223	74	hsa-miR-21-3p	PCDHA10	protocadherin alpha 10
224	74	hsa-miR-21-3p	PCDHAC2	protocadherin alpha subfamily C, 2
225	74	hsa-miR-21-3p	ZNF302	zinc finger protein 302
226	74	hsa-miR-21-3p	PNPLA8	patatin like phospholipase domain containing 8
227	74	hsa-miR-21-3p	PCDHA5	protocadherin alpha 5
228	74	hsa-miR-21-3p	MIB1	mindbomb E3 ubiquitin protein ligase 1
229	73	hsa-miR-21-3p	SIRPA	signal regulatory protein alpha
230	73	hsa-miR-21-3p	NT5C1B-RDH14	NT5C1B-RDH14 readthrough
231	73	hsa-miR-21-3p	DTL	denticleless E3 ubiquitin protein ligase homolog
232	73	hsa-miR-21-3p	IPO5	importin 5
233	73	hsa-miR-21-3p	GAREM1	GRB2 associated regulator of MAPK1 subtype 1
234	73	hsa-miR-21-3p	TSC1	TSC complex subunit 1
235	73	hsa-miR-21-3p	UHRF2	ubiquitin like with PHD and ring finger domains 2
236	73	hsa-miR-21-3p	MAPK1	mitogen-activated protein kinase 1
237	73	hsa-miR-21-3p	ICOS	inducible T cell costimulator
238	73	hsa-miR-21-3p	RDH14	retinol dehydrogenase 14
239	73	hsa-miR-21-3p	TNN	tenascin N
240	73	hsa-miR-21-3p	SHISA7	shisa family member 7
241	72	hsa-miR-21-3p	SEMA6A	semaphorin 6A
242	72	hsa-miR-21-3p	ZNF609	zinc finger protein 609
243	72	hsa-miR-21-3p	ADTRP	androgen dependent TFPI regulating protein
244	72	hsa-miR-21-3p	CDH11	cadherin 11
245	72	hsa-miR-21-3p	CDK6	cyclin dependent kinase 6
246	72	hsa-miR-21-3p	GIMAP6	GTPase, IMAP family member 6
247	72	hsa-miR-21-3p	PREP	prolyl endopeptidase
248	72	hsa-miR-21-3p	ZNF891	zinc finger protein 891
249	72	hsa-miR-21-3p	SRSF1	serine and arginine rich splicing factor 1
250	72	hsa-miR-21-3p	DNAJC15	DnaJ heat shock protein family (Hsp40) member C15
251	72	hsa-miR-21-3p	GRID1	glutamate ionotropic receptor delta type subunit 1
252	72	hsa-miR-21-3p	ZNF200	zinc finger protein 200
253	71	hsa-miR-21-3p	IPCEF1	interaction protein for cytohesin exchange factors 1
254	71	hsa-miR-21-3p	RPS6KA2	ribosomal protein S6 kinase A2
255	71	hsa-miR-21-3p	CTPS1	CTP synthase 1
256	71	hsa-miR-21-3p	USP45	ubiquitin specific peptidase 45
257	71	hsa-miR-21-3p	GABRB3	gamma-aminobutyric acid type A receptor beta3 subunit
258	71	hsa-miR-21-3p	AHCYL1	adenosylhomocysteinase like 1
259	71	hsa-miR-21-3p	CLCN3	chloride voltage-gated channel 3
260	71	hsa-miR-21-3p	PPFIBP1	PPFIA binding protein 1
261	71	hsa-miR-21-3p	PLGLB1	plasminogen-like B1
262	71	hsa-miR-21-3p	APOPT1	apoptogenic 1, mitochondrial

263	71	hsa-miR-21-3p	PLGLB2	plasminogen-like B2
264	71	hsa-miR-21-3p	CHST3	carbohydrate sulfotransferase 3
265	71	hsa-miR-21-3p	IGFBP4	insulin like growth factor binding protein 4
266	71	hsa-miR-21-3p	MON2	MON2 homolog, regulator of endosome-to-Golgi trafficking
267	70	hsa-miR-21-3p	SLC9A7	solute carrier family 9 member A7
268	70	hsa-miR-21-3p	CCAR1	cell division cycle and apoptosis regulator 1
269	70	hsa-miR-21-3p	UBXN7	UBX domain protein 7
270	70	hsa-miR-21-3p	PHYHD1	phytanoyl-CoA dioxygenase domain containing 1
271	70	hsa-miR-21-3p	RBPMS	RNA binding protein, mRNA processing factor
272	70	hsa-miR-21-3p	GALNT15	polypeptide N-acetylgalactosaminyltransferase 15
273	70	hsa-miR-21-3p	ELK4	ELK4, ETS transcription factor
274	70	hsa-miR-21-3p	BCL11A	BCL11A, BAF complex component
275	69	hsa-miR-21-3p	CHORDC1	cysteine and histidine rich domain containing 1
276	69	hsa-miR-21-3p	GNAQ	G protein subunit alpha q
277	69	hsa-miR-21-3p	AMIGO2	adhesion molecule with Ig like domain 2
278	69	hsa-miR-21-3p	TMX4	thioredoxin related transmembrane protein 4
279	69	hsa-miR-21-3p	PRPS2	phosphoribosyl pyrophosphate synthetase 2
280	69	hsa-miR-21-3p	MAFB	MAF bZIP transcription factor B
281	69	hsa-miR-21-3p	PRPF40B	pre-mRNA processing factor 40 homolog B
282	69	hsa-miR-21-3p	RCBTB1	RCC1 and BTB domain containing protein 1
283	69	hsa-miR-21-3p	INO80D	INO80 complex subunit D
284	69	hsa-miR-21-3p	CYLD	CYLD lysine 63 deubiquitinase
285	69	hsa-miR-21-3p	L2HGDH	L-2-hydroxyglutarate dehydrogenase
286	69	hsa-miR-21-3p	FASN	fatty acid synthase
287	69	hsa-miR-21-3p	ZFHX3	zinc finger homeobox 3
288	69	hsa-miR-21-3p	ZNF621	zinc finger protein 621
289	68	hsa-miR-21-3p	CUL5	cullin 5
290	68	hsa-miR-21-3p	GJA1	gap junction protein alpha 1
291	68	hsa-miR-21-3p	ITPRID2	ITPR interacting domain containing 2
292	68	hsa-miR-21-3p	SV2C	synaptic vesicle glycoprotein 2C
293	68	hsa-miR-21-3p	ZNF250	zinc finger protein 250
294	68	hsa-miR-21-3p	NUFIP2	nuclear FMR1 interacting protein 2
295	68	hsa-miR-21-3p	FRS2	fibroblast growth factor receptor substrate 2
296	68	hsa-miR-21-3p	CLTC	clathrin heavy chain
297	68	hsa-miR-21-3p	WWC2	WW and C2 domain containing 2
298	68	hsa-miR-21-3p	MDM4	MDM4, p53 regulator
299	68	hsa-miR-21-3p	PACRGL	parkin coregulated like
300	68	hsa-miR-21-3p	SEMA3E	semaphorin 3E
301	68	hsa-miR-21-3p	TMEM170B	transmembrane protein 170B
302	68	hsa-miR-21-3p	MTA2	metastasis associated 1 family member 2
303	68	hsa-miR-21-3p	ST6GALNAC5	ST6 N-acetylgalactosaminide alpha-2,6-sialyltransferase 5
304	68	hsa-miR-21-3p	NCALD	neurocalcin delta
305	68	hsa-miR-21-3p	ZNF101	zinc finger protein 101
306	68	hsa-miR-21-3p	YPEL5	yippee like 5
307	68	hsa-miR-21-3p	RBM33	RNA binding motif protein 33
308	68	hsa-miR-21-3p	TMEM56-RWDD3	TMEM56-RWDD3 readthrough
309	68	hsa-miR-21-3p	GFRA1	GDNF family receptor alpha 1
310	67	hsa-miR-21-3p	NIPA1	NIPA magnesium transporter 1
311	67	hsa-miR-21-3p	PLXDC2	plexin domain containing 2
312	67	hsa-miR-21-3p	LITAF	lipopolysaccharide induced TNF factor
313	67	hsa-miR-21-3p	CBLB	Cbl proto-oncogene B
314	67	hsa-miR-21-3p	TRIQK	triple QxxK/R motif containing
315	67	hsa-miR-21-3p	BAALC	BAALC, MAP3K1 and KLF4 binding
316	67	hsa-miR-21-3p	TFRC	transferrin receptor

317	67	hsa-miR-21-3p	SDC2	syndecan 2
318	67	hsa-miR-21-3p	ARNTL	aryl hydrocarbon receptor nuclear translocator like
319	67	hsa-miR-21-3p	MAP3K7	mitogen-activated protein kinase kinase kinase 7
320	67	hsa-miR-21-3p	GRHL1	grainyhead like transcription factor 1
321	67	hsa-miR-21-3p	S1PR3	sphingosine-1-phosphate receptor 3
322	67	hsa-miR-21-3p	B4GALT6	beta-1,4-galactosyltransferase 6
323	66	hsa-miR-21-3p	POFUT2	protein O-fucosyltransferase 2
324	66	hsa-miR-21-3p	PRPF38B	pre-mRNA processing factor 38B
325	66	hsa-miR-21-3p	DIO2	iodothyronine deiodinase 2
326	66	hsa-miR-21-3p	OLFM3	olfactomedin 3
327	66	hsa-miR-21-3p	SCYL2	SCY1 like pseudokinase 2
328	66	hsa-miR-21-3p	TEAD1	TEA domain transcription factor 1
329	66	hsa-miR-21-3p	AGGF1	angiogenic factor with G-patch and FHA domains 1
330	66	hsa-miR-21-3p	RSRC2	arginine and serine rich coiled-coil 2
331	66	hsa-miR-21-3p	SNN	stannin
332	66	hsa-miR-21-3p	LRRC17	leucine rich repeat containing 17
333	66	hsa-miR-21-3p	TMEM47	transmembrane protein 47
334	66	hsa-miR-21-3p	VPS13C	vacuolar protein sorting 13 homolog C
335	65	hsa-miR-21-3p	DISC1	DISC1 scaffold protein
336	65	hsa-miR-21-3p	SLC11A2	solute carrier family 11 member 2
337	65	hsa-miR-21-3p	MAP3K9	mitogen-activated protein kinase kinase kinase 9
338	65	hsa-miR-21-3p	CCDC32	coiled-coil domain containing 32
339	65	hsa-miR-21-3p	SCAI	suppressor of cancer cell invasion
340	65	hsa-miR-21-3p	FKBP3	FKBP prolyl isomerase 3
341	65	hsa-miR-21-3p	KCNK10	potassium two pore domain channel subfamily K member 10
342	65	hsa-miR-21-3p	EML4	EMAP like 4
343	65	hsa-miR-21-3p	AKAIN1	A-kinase anchor inhibitor 1
344	65	hsa-miR-21-3p	POU4F2	POU class 4 homeobox 2
345	65	hsa-miR-21-3p	KCTD7	potassium channel tetramerization domain containing 7
346	65	hsa-miR-21-3p	MAP1B	microtubule associated protein 1B
347	65	hsa-miR-21-3p	C9orf47	chromosome 9 open reading frame 47
348	65	hsa-miR-21-3p	PTPRO	protein tyrosine phosphatase, receptor type O
349	65	hsa-miR-21-3p	TBC1D24	TBC1 domain family member 24
350	65	hsa-miR-21-3p	PKD1	polycystin 1, transient receptor potential channel interacting
351	65	hsa-miR-21-3p	RNF150	ring finger protein 150
352	65	hsa-miR-21-3p	CAMLG	calcium modulating ligand
353	65	hsa-miR-21-3p	AMD1	adenosylmethionine decarboxylase 1
354	65	hsa-miR-21-3p	ST3GAL1	ST3 beta-galactoside alpha-2,3-sialyltransferase 1
355	64	hsa-miR-21-3p	ZNRF1	zinc and ring finger 1
356	64	hsa-miR-21-3p	SEMA6D	semaphorin 6D
357	64	hsa-miR-21-3p	RLIM	ring finger protein, LIM domain interacting
358	64	hsa-miR-21-3p	SRSF8	serine and arginine rich splicing factor 8
359	64	hsa-miR-21-3p	ETV1	ETS variant 1
360	64	hsa-miR-21-3p	PJVK	pejvakin
361	64	hsa-miR-21-3p	SLC25A32	solute carrier family 25 member 32
362	64	hsa-miR-21-3p	RORB	RAR related orphan receptor B
363	64	hsa-miR-21-3p	HRH1	histamine receptor H1
364	64	hsa-miR-21-3p	ENTPD4	ectonucleoside triphosphate diphosphohydrolase 4
365	64	hsa-miR-21-3p	PIK3IP1	phosphoinositide-3-kinase interacting protein 1
366	64	hsa-miR-21-3p	ZMAT3	zinc finger matrin-type 3
367	63	hsa-miR-21-3p	C1GALT1	core 1 synthase, glycoprotein-N-acetylgalactosamine 3-beta-galactosyltransferase 1
368	63	hsa-miR-21-3p	SH3TC2	SH3 domain and tetratricopeptide repeats 2
369	63	hsa-miR-21-3p	IL6R	interleukin 6 receptor

370	63	hsa-miR-21-3p	ME2	malic enzyme 2
371	63	hsa-miR-21-3p	DYNC1LI2	dynein cytoplasmic 1 light intermediate chain 2
372	63	hsa-miR-21-3p	HOXC11	homeobox C11
373	63	hsa-miR-21-3p	CBY1	chibby family member 1, beta catenin antagonist
374	63	hsa-miR-21-3p	HOOK3	hook microtubule tethering protein 3
375	63	hsa-miR-21-3p	IL22RA2	interleukin 22 receptor subunit alpha 2
376	63	hsa-miR-21-3p	VIP	vasoactive intestinal peptide
377	63	hsa-miR-21-3p	DYNC1I1	dynein cytoplasmic 1 intermediate chain 1
378	63	hsa-miR-21-3p	EML2	EMAP like 2
379	63	hsa-miR-21-3p	RHD	Rh blood group D antigen
380	63	hsa-miR-21-3p	RIC1	RIC1 homolog, RAB6A GEF complex partner 1
381	63	hsa-miR-21-3p	ACAD11	acyl-CoA dehydrogenase family member 11
382	63	hsa-miR-21-3p	CCDC184	coiled-coil domain containing 184
383	63	hsa-miR-21-3p	PAX6	paired box 6
384	63	hsa-miR-21-3p	TBX18	T-box 18
385	62	hsa-miR-21-3p	FRMPD4	FERM and PDZ domain containing 4
386	62	hsa-miR-21-3p	CCR6	C-C motif chemokine receptor 6
387	62	hsa-miR-21-3p	SIRPB1	signal regulatory protein beta 1
388	62	hsa-miR-21-3p	RASEF	RAS and EF-hand domain containing
389	62	hsa-miR-21-3p	AKAP6	A-kinase anchoring protein 6
390	62	hsa-miR-21-3p	F3	coagulation factor III, tissue factor
391	62	hsa-miR-21-3p	SCRIB	scribbled planar cell polarity protein
392	62	hsa-miR-21-3p	DUXA	double homeobox A
393	62	hsa-miR-21-3p	PGS1	phosphatidylglycerophosphate synthase 1
394	62	hsa-miR-21-3p	TRIM22	tripartite motif containing 22
395	62	hsa-miR-21-3p	PTGES3	prostaglandin E synthase 3
396	62	hsa-miR-21-3p	ZC3H6	zinc finger CCCH-type containing 6
397	62	hsa-miR-21-3p	MEF2A	myocyte enhancer factor 2A
398	62	hsa-miR-21-3p	FPR2	formyl peptide receptor 2
399	62	hsa-miR-21-3p	FAXC	failed axon connections homolog
400	62	hsa-miR-21-3p	FAM124B	family with sequence similarity 124 member B
401	62	hsa-miR-21-3p	CNTNAP4	contactin associated protein like 4
402	62	hsa-miR-21-3p	ARL2BP	ADP ribosylation factor like GTPase 2 binding protein
403	61	hsa-miR-21-3p	HOOK1	hook microtubule tethering protein 1
404	61	hsa-miR-21-3p	PGAP1	post-GPI attachment to proteins 1
405	61	hsa-miR-21-3p	TXNRD2	thioredoxin reductase 2
406	61	hsa-miR-21-3p	TSHZ1	teashirt zinc finger homeobox 1
407	61	hsa-miR-21-3p	SLC25A53	solute carrier family 25 member 53
408	61	hsa-miR-21-3p	TLDC2	TBC/LysM-associated domain containing 2
409	61	hsa-miR-21-3p	MYOZ2	myozenin 2
410	61	hsa-miR-21-3p	FRYL	FRY like transcription coactivator
411	61	hsa-miR-21-3p	PURG	purine rich element binding protein G
412	61	hsa-miR-21-3p	KIF5B	kinesin family member 5B
413	61	hsa-miR-21-3p	PRKAR2A	protein kinase cAMP-dependent type II regulatory subunit alpha
414	61	hsa-miR-21-3p	NPR3	natriuretic peptide receptor 3
415	60	hsa-miR-21-3p	FTO	FTO, alpha-ketoglutarate dependent dioxygenase
416	60	hsa-miR-21-3p	CACNB4	calcium voltage-gated channel auxiliary subunit beta 4
417	60	hsa-miR-21-3p	SLC25A37	solute carrier family 25 member 37
418	60	hsa-miR-21-3p	CGGBP1	CGG triplet repeat binding protein 1
419	60	hsa-miR-21-3p	KCNK9	potassium two pore domain channel subfamily K member 9
420	60	hsa-miR-21-3p	LSAMP	limbic system associated membrane protein
421	60	hsa-miR-21-3p	BANP	BTG3 associated nuclear protein
422	60	hsa-miR-21-3p	ATE1	arginyltransferase 1
423	60	hsa-miR-21-3p	GPATCH2	G-patch domain containing 2

424	60	hsa-miR-21-3p	HAL	histidine ammonia-lyase
425	60	hsa-miR-21-3p	PRPF4B	pre-mRNA processing factor 4B
426	60	hsa-miR-21-3p	USP22	ubiquitin specific peptidase 22
427	60	hsa-miR-21-3p	M6PR	mannose-6-phosphate receptor, cation dependent
428	59	hsa-miR-21-3p	PHF20L1	PHD finger protein 20 like 1
429	59	hsa-miR-21-3p	TRA2B	transformer 2 beta homolog
430	59	hsa-miR-21-3p	GNAZ	G protein subunit alpha z
431	59	hsa-miR-21-3p	ABI2	abl interactor 2
432	59	hsa-miR-21-3p	RAB18	RAB18, member RAS oncogene family
433	59	hsa-miR-21-3p	CNIH3	cornichon family AMPA receptor auxiliary protein 3
434	59	hsa-miR-21-3p	DCUN1D5	defective in cullin neddylation 1 domain containing 5
435	59	hsa-miR-21-3p	TMEM241	transmembrane protein 241
436	59	hsa-miR-21-3p	KCMF1	potassium channel modulatory factor 1
437	59	hsa-miR-21-3p	ANO5	anoctamin 5
438	59	hsa-miR-21-3p	DSG1	desmoglein 1
439	59	hsa-miR-21-3p	PHF8	PHD finger protein 8
440	59	hsa-miR-21-3p	MYBPH	myosin binding protein H
441	58	hsa-miR-21-3p	BMP3	bone morphogenetic protein 3
442	58	hsa-miR-21-3p	HNRNPR	heterogeneous nuclear ribonucleoprotein R
443	58	hsa-miR-21-3p	MYOZ3	myozenin 3
444	58	hsa-miR-21-3p	SAR1A	secretion associated Ras related GTPase 1A
445	58	hsa-miR-21-3p	FBXO11	F-box protein 11
446	58	hsa-miR-21-3p	MIA3	MIA SH3 domain ER export factor 3
447	58	hsa-miR-21-3p	UNC5D	unc-5 netrin receptor D
448	58	hsa-miR-21-3p	FGF14	fibroblast growth factor 14
449	58	hsa-miR-21-3p	CCDC158	coiled-coil domain containing 158
450	58	hsa-miR-21-3p	DPP8	dipeptidyl peptidase 8
451	58	hsa-miR-21-3p	B4GALT1	beta-1,4-galactosyltransferase 1
452	58	hsa-miR-21-3p	RBMS3	RNA binding motif single stranded interacting protein 3
453	57	hsa-miR-21-3p	STIM2	stromal interaction molecule 2
454	57	hsa-miR-21-3p	WWC1	WW and C2 domain containing 1
455	57	hsa-miR-21-3p	PRR14L	proline rich 14 like
456	57	hsa-miR-21-3p	HLCS	holocarboxylase synthetase
457	57	hsa-miR-21-3p	ABRA	actin binding Rho activating protein
458	57	hsa-miR-21-3p	KLHL7	kelch like family member 7
459	57	hsa-miR-21-3p	IMPA2	inositol monophosphatase 2
460	57	hsa-miR-21-3p	POU3F3	POU class 3 homeobox 3
461	57	hsa-miR-21-3p	EPB41L4A	erythrocyte membrane protein band 4.1 like 4A
462	57	hsa-miR-21-3p	SNRPB2	small nuclear ribonucleoprotein polypeptide B2
463	57	hsa-miR-21-3p	ZDHHC8	zinc finger DHHC-type containing 8
464	57	hsa-miR-21-3p	ELOVL4	ELOVL fatty acid elongase 4
465	57	hsa-miR-21-3p	DAXX	death domain associated protein
466	56	hsa-miR-21-3p	PDE4D	phosphodiesterase 4D
467	56	hsa-miR-21-3p	TPD52L3	TPD52 like 3
468	56	hsa-miR-21-3p	CALM1	calmodulin 1
469	56	hsa-miR-21-3p	SNIP1	Smad nuclear interacting protein 1
470	56	hsa-miR-21-3p	CLIP1	CAP-Gly domain containing linker protein 1
471	56	hsa-miR-21-3p	OTUD3	OTU deubiquitinase 3
472	56	hsa-miR-21-3p	CFAP20	cilia and flagella associated protein 20
473	56	hsa-miR-21-3p	CD80	CD80 molecule
474	56	hsa-miR-21-3p	SLC44A1	solute carrier family 44 member 1
475	56	hsa-miR-21-3p	KIAA1147	KIAA1147
476	56	hsa-miR-21-3p	PASK	PAS domain containing serine/threonine kinase
477	56	hsa-miR-21-3p	ATG10	autophagy related 10

478	55	hsa-miR-21-3p	ST6GAL2	ST6 beta-galactoside alpha-2,6-sialyltransferase 2
479	55	hsa-miR-21-3p	NEK9	NIMA related kinase 9
480	55	hsa-miR-21-3p	MLH3	mutL homolog 3
481	55	hsa-miR-21-3p	ATRNL1	attractin like 1
482	55	hsa-miR-21-3p	POLR2C	RNA polymerase II subunit C
483	55	hsa-miR-21-3p	FBP1	fructose-bisphosphatase 1
484	55	hsa-miR-21-3p	TBL1XR1	transducin beta like 1 X-linked receptor 1
485	55	hsa-miR-21-3p	ENDOV	endonuclease V
486	55	hsa-miR-21-3p	ADCK1	aarF domain containing kinase 1
487	55	hsa-miR-21-3p	ZNF697	zinc finger protein 697
488	55	hsa-miR-21-3p	SYNPO2	synaptopodin 2
489	55	hsa-miR-21-3p	CCSER2	coiled-coil serine rich protein 2
490	55	hsa-miR-21-3p	SRRM4	serine/arginine repetitive matrix 4
491	55	hsa-miR-21-3p	TRIM32	tripartite motif containing 32
492	55	hsa-miR-21-3p	SRGAP2	SLIT-ROBO Rho GTPase activating protein 2
493	55	hsa-miR-21-3p	NT5C1B	5'-nucleotidase, cytosolic IB
494	55	hsa-miR-21-3p	MMRN2	multimerin 2
495	54	hsa-miR-21-3p	TET2	tet methylcytosine dioxygenase 2
496	54	hsa-miR-21-3p	BMP6	bone morphogenetic protein 6
497	54	hsa-miR-21-3p	ATRN	attractin
498	54	hsa-miR-21-3p	OTUD4	OTU deubiquitinase 4
499	54	hsa-miR-21-3p	ZNF217	zinc finger protein 217
500	54	hsa-miR-21-3p	RBM12	RNA binding motif protein 12
501	54	hsa-miR-21-3p	CBL	Cbl proto-oncogene
502	54	hsa-miR-21-3p	SAP30L	SAP30 like
503	54	hsa-miR-21-3p	ZNF831	zinc finger protein 831
504	54	hsa-miR-21-3p	CDH7	cadherin 7
505	54	hsa-miR-21-3p	EPB41L5	erythrocyte membrane protein band 4.1 like 5
506	54	hsa-miR-21-3p	SSPN	sarcospan
507	54	hsa-miR-21-3p	MTF2	metal response element binding transcription factor 2
508	54	hsa-miR-21-3p	H3F3A	H3 histone family member 3A
509	54	hsa-miR-21-3p	QSER1	glutamine and serine rich 1
510	53	hsa-miR-21-3p	SLC9A4	solute carrier family 9 member A4
511	53	hsa-miR-21-3p	GPD2	glycerol-3-phosphate dehydrogenase 2
512	53	hsa-miR-21-3p	PSMD11	proteasome 26S subunit, non-ATPase 11
513	53	hsa-miR-21-3p	GTF2A1	general transcription factor IIA subunit 1
514	53	hsa-miR-21-3p	HBP1	HMG-box transcription factor 1
515	53	hsa-miR-21-3p	INSIG1	insulin induced gene 1
516	53	hsa-miR-21-3p	DUT	deoxyuridine triphosphatase
517	53	hsa-miR-21-3p	CHIC1	cysteine rich hydrophobic domain 1
518	53	hsa-miR-21-3p	ADGRB3	adhesion G protein-coupled receptor B3
519	53	hsa-miR-21-3p	ECM2	extracellular matrix protein 2
520	53	hsa-miR-21-3p	TBX5	T-box 5
521	53	hsa-miR-21-3p	DCLK1	doublecortin like kinase 1
522	53	hsa-miR-21-3p	CIP2A	cell proliferation regulating inhibitor of protein phosphatase 2A
523	53	hsa-miR-21-3p	CHL1	cell adhesion molecule L1 like
524	53	hsa-miR-21-3p	INSM2	INSM transcriptional repressor 2
525	53	hsa-miR-21-3p	C12orf4	chromosome 12 open reading frame 4
526	53	hsa-miR-21-3p	MTREX	Mtr4 exosome RNA helicase
527	53	hsa-miR-21-3p	LSM4	LSM4 homolog, U6 small nuclear RNA and mRNA degradation associated
528	53	hsa-miR-21-3p	TMOD3	tropomodulin 3
529	53	hsa-miR-21-3p	NSD3	nuclear receptor binding SET domain protein 3
530	52	hsa-miR-21-3p	OCIAD1	OCIA domain containing 1

531	52	hsa-miR-21-3p	BCAT1	branched chain amino acid transaminase 1
532	52	hsa-miR-21-3p	SOX4	SRY-box 4
533	52	hsa-miR-21-3p	IGF2R	insulin like growth factor 2 receptor
534	52	hsa-miR-21-3p	MARVELD3	MARVEL domain containing 3
535	52	hsa-miR-21-3p	USP46	ubiquitin specific peptidase 46
536	52	hsa-miR-21-3p	RBM14	RNA binding motif protein 14
537	52	hsa-miR-21-3p	MYRF	myelin regulatory factor
538	52	hsa-miR-21-3p	RAB3C	RAB3C, member RAS oncogene family
539	52	hsa-miR-21-3p	TP53	tumor protein p53
540	52	hsa-miR-21-3p	DOCK10	dedicator of cytokinesis 10
541	52	hsa-miR-21-3p	SUSD6	sushi domain containing 6
542	52	hsa-miR-21-3p	GNB4	G protein subunit beta 4
543	52	hsa-miR-21-3p	ZNF225	zinc finger protein 225
544	52	hsa-miR-21-3p	ILF2	interleukin enhancer binding factor 2
545	52	hsa-miR-21-3p	ZNF805	zinc finger protein 805
546	51	hsa-miR-21-3p	UBE2L3	ubiquitin conjugating enzyme E2 L3
547	51	hsa-miR-21-3p	ARHGAP36	Rho GTPase activating protein 36
548	51	hsa-miR-21-3p	ATP13A3	ATPase 13A3
549	51	hsa-miR-21-3p	POLR3A	RNA polymerase III subunit A
550	51	hsa-miR-21-3p	PPARGC1B	PPARG coactivator 1 beta
551	51	hsa-miR-21-3p	B3GNT5	UDP-GlcNAc:betaGal beta-1,3-N-acetylglucosaminyltransferase 5
552	51	hsa-miR-21-3p	CDK15	cyclin dependent kinase 15
553	51	hsa-miR-21-3p	TAX1BP1	Tax1 binding protein 1
554	51	hsa-miR-21-3p	ARHGAP6	Rho GTPase activating protein 6
555	51	hsa-miR-21-3p	RIMS2	regulating synaptic membrane exocytosis 2
556	51	hsa-miR-21-3p	FBXO42	F-box protein 42
557	51	hsa-miR-21-3p	SRSF7	serine and arginine rich splicing factor 7
558	51	hsa-miR-21-3p	LIN28B	lin-28 homolog B
559	51	hsa-miR-21-3p	RNF168	ring finger protein 168
560	51	hsa-miR-21-3p	LAMP2	lysosomal associated membrane protein 2
561	51	hsa-miR-21-3p	TIAL1	TIA1 cytotoxic granule associated RNA binding protein like 1
562	51	hsa-miR-21-3p	EEF1A1	eukaryotic translation elongation factor 1 alpha 1
563	51	hsa-miR-21-3p	DYRK1A	dual specificity tyrosine phosphorylation regulated kinase 1A
564	51	hsa-miR-21-3p	WDR92	WD repeat domain 92
565	51	hsa-miR-21-3p	NFATC2	nuclear factor of activated T cells 2
566	51	hsa-miR-21-3p	SIAH3	siah E3 ubiquitin protein ligase family member 3
567	51	hsa-miR-21-3p	FMNL3	formin like 3
568	51	hsa-miR-21-3p	EIF2S1	eukaryotic translation initiation factor 2 subunit alpha
569	51	hsa-miR-21-3p	CEBPG	CCAAT enhancer binding protein gamma
570	51	hsa-miR-21-3p	CCSER1	coiled-coil serine rich protein 1
571	50	hsa-miR-21-3p	PLAGL2	PLAG1 like zinc finger 2
572	50	hsa-miR-21-3p	B3GNT7	UDP-GlcNAc:betaGal beta-1,3-N-acetylglucosaminyltransferase 7
573	50	hsa-miR-21-3p	TTL	tubulin tyrosine ligase
574	50	hsa-miR-21-3p	CTNNB1	catenin beta 1
575	50	hsa-miR-21-3p	SMAD1	SMAD family member 1
576	50	hsa-miR-21-3p	EPM2AIP1	EPM2A interacting protein 1
577	50	hsa-miR-21-3p	PSMF1	proteasome inhibitor subunit 1
578	50	hsa-miR-21-3p	HNRNPH1	heterogeneous nuclear ribonucleoprotein H1
579	50	hsa-miR-21-3p	CR2	complement C3d receptor 2
580	50	hsa-miR-21-3p	ASH1L	ASH1 like histone lysine methyltransferase
581	50	hsa-miR-21-3p	THUMPD3	THUMP domain containing 3
582	50	hsa-miR-21-3p	RNASEL	ribonuclease L
583	50	hsa-miR-21-3p	TBRG4	transforming growth factor beta regulator 4
584	50	hsa-miR-21-3p	DEK	DEK proto-oncogene

585	50	hsa-miR-21-3p	SEC14L5	SEC14 like lipid binding 5
586	50	hsa-miR-21-3p	PHF2	PHD finger protein 2
587	50	hsa-miR-21-3p	MAB21L1	mab-21 like 1
588	50	hsa-miR-21-3p	TOM1	target of myb1 membrane trafficking protein
589	50	hsa-miR-21-3p	MTF1	metal regulatory transcription factor 1
590	50	hsa-miR-21-3p	NCOA3	nuclear receptor coactivator 3
591	50	hsa-miR-21-3p	CDCA7	cell division cycle associated 7
592	50	hsa-miR-21-3p	CASP7	caspase 7
593	50	hsa-miR-21-3p	CNRIP1	cannabinoid receptor interacting protein 1
594	50	hsa-miR-21-3p	ZBTB21	zinc finger and BTB domain containing 21
595	50	hsa-miR-21-3p	ZNF264	zinc finger protein 264

Table 4. Toxicity functions reported for hsa-miR-21-5p. Cardiotoxicity functions identified via Ingenuity Pathway Analysis of all target genes of hsa-miR-21-5p are reported.

Category	Molecules
Liver Proliferation	FASLG,SKP2,YOD1
Cardiac Dilation	VCL
Cardiac Enlargement	FASLG,VCL
Heart Failure	RBPJ,VCL
Cardiac Hypoplasia	PBRM1,RBPJ
Cardiac Dysfunction	FASLG,RBPJ,VCL
Degeneration of liver	FASLG
Liver Necrosis/Cell Death	FASLG
Liver Enlargement	FASLG,YOD1
Liver Cirrhosis	FASLG,IL12A
Liver Failure	FASLG
Glutathione Depletion In Liver	FASLG
Liver Fibrosis	FASLG
Renal Proliferation	SKP2
Liver Inflammation/Hepatitis	FASLG
Cardiac Arrythmia	VCL
Increased Levels of AST	FASLG
Liver Hyperplasia/ Hyperproliferation	FASLG,PBRM1,RALGPS2,RBPJ,SKP2,TGFBI,VCL,YOD1
Renal Necrosis/Cell Death	FASLG
Glomerular Injury	FASLG,SKP2
Liver Damage	FASLG
Cardiac Inflammation	RBPJ
Kidney Failure	SKP2
Renal Fibrosis	SKP2
Tachycardia	VCL
Cardiac Proliferation	RBPJ
Cardiac Necrosis/Cell Death	FASLG
Cardiac Fibrosis	RBPJ
Cardiac Infarction	FASLG
Renal Inflammation	FASLG
Renal Nephritis	FASLG
Liver Steatosis	RBPJ

Table 5. Toxicity functions reported for hsa-miR-21-3p. Cardiotoxicity functions identified via Ingenuity Pathway Analysis of all target genes of hsa-miR-21-3p are reported.

Category	Molecules
Cardiac Necrosis/Cell Death	FOXO3,MAP2K4,MAP3K1,UBE4B
Heart Failure	MAP2K4,MAP3K1,UBE4B
Liver Regeneration	MAP2K4
Cardiac Congestive Cardiac Failure	MAP3K1,UBE4B
Kidney Failure	ATP1B1
Renal Damage	ATP1B1
Cardiac Enlargement	FOXO3,MAP2K4,UBE4B
Cardiac Arrythmia	ATP1B1,FOXO3
Liver Proliferation	MAP2K4
Liver Necrosis/Cell Death	FOXO3,MAP2K4
Liver Hyperplasia/Hyperproliferation	AKAP11,ALCAM,ATP1B1,CDK8,GPM6A,GRIA2,MAP2K4,MAP3K1, PARD3B, PCDH19,TOGARAM1
Liver Hemorrhaging	MAP2K4
Renal Necrosis/Cell Death	MAP2K4,MAP3K1
Liver Damage	PARD3B
Cardiac Inflammation	MAP3K1
Increased Levels of Alkaline Phosphatase	MAP3K1
Liver Failure	MAP2K4
Renal Hydronephrosis	ALCAM
Increased Levels of Red Blood Cells	FOXO3
Glomerular Injury	ATP1B1
Renal Fibrosis	ATP1B1
Renal Proliferation	FOXO3
Liver Cirrhosis	ATP1B1
Cardiac Infarction	FOXO3
Cardiac Dilation	FOXO3
Liver Steatosis	FOXO3
Hepatocellular carcinoma	ATP1B1,GPM6A
Renal Inflammation	FOXO3
Renal Nephritis	FOXO3
